# An Integrative Analysis Reveals a Central Role of P53 Activation via MDM2 in Zika Virus Infection Induced Cell Death

**DOI:** 10.3389/fcimb.2017.00327

**Published:** 2017-07-20

**Authors:** Yue Teng, Shufeng Liu, Xiaocan Guo, Shuxia Liu, Yuan Jin, Tongtong He, Dehua Bi, Pei Zhang, Baihan Lin, Xiaoping An, Dan Feng, Zhiqiang Mi, Yigang Tong

**Affiliations:** ^1^State Key Laboratory of Pathogen and Biosecurity Beijing, China; ^2^Beijing Institute of Microbiology and Epidemiology Beijing, China; ^3^Center for Infectious Diseases, SRI International Harrisonburg, VA, United States; ^4^Massachusetts Institute of Technology Cambridge, MA, United States; ^5^College of Nuclear Science and Technology, Beijing Normal University Beijing, China; ^6^Beijing Institute of Biotechnology Beijing, China; ^7^Department of Neurobiology, Tongji Medical School, Huazhong University of Science and Technology Wuhan, China; ^8^Computational Neuroscience Program, Department of Psychology, Physics, and Computer Science and Engineering; Institute for Protein Design, University of Washington Seattle, WA, United States; ^9^Division of Standard Operational Management, Institute of Hospital Management, Chinese PLA General Hospital Beijing, China

**Keywords:** *Zika virus*, microcephaly, capsid protein, P53, MDM2, cell death

## Abstract

*Zika virus* (ZIKV) infection is an emerging global threat that is suspected to be associated with fetal microcephaly. However, the molecular mechanisms underlying ZIKV disease pathogenesis in humans remain elusive. Here, we investigated the human protein interaction network associated with ZIKV infection using a systemic virology approach, and reconstructed the transcriptional regulatory network to analyze the mechanisms underlying ZIKV-elicited microcephaly pathogenesis. The bioinformatics findings in this study show that P53 is the hub of the genetic regulatory network for ZIKV-related and microcephaly-associated proteins. Importantly, these results imply that the ZIKV capsid protein interacts with mouse double-minute-2 homolog (MDM2), which is involved in the P53-mediated apoptosis pathway, activating the death of infected neural cells. We also found that synthetic mimics of the ZIKV capsid protein induced cell death *in vitro* and *in vivo*. This study provides important insight into the relationship between ZIKV infection and brain diseases.

## Introduction

*Zika virus* (ZIKV) is a single-stranded, positive-sense RNA virus belonging to the genus *Flavivirus* in the family *Flaviviridae*, which also includes *Dengue virus, West Nile virus, Japanese encephalitis virus*, and *Yellow fever virus* (Musso and Gubler, 2016; Weaver et al., 2016). Zika virus diseases (ZVD) are caused by ZIKV, an emerging mosquito-borne virus, and have threatened to become a global pandemic since 2015 (Fauci and Morens, 2016; Petersen et al., 2016). Epidemiological evidence suggests that ZIKV infection in pregnant women in Brazil is associated with the increasing numbers of congenital microcephaly cases reported in that country (Calvet et al., 2016; Cauchemez et al., 2016; Gabriel et al., 2017). However, a direct causal link between ZIKV and microcephaly has yet to be demonstrated (Cugola et al., 2016; Li et al., 2016; Miner et al., 2016). There is currently no vaccine or drug to prevent or treat ZIKV infection (Mlakar et al., 2016; Rasmussen et al., 2016).

Targeting the molecular factors in human cells that are related to ZVD is a potentially useful strategy for the development of therapeutic drugs for these conditions (Hamel et al., 2015). Biomarkers are also urgently required to verify the relationship between ZIKV infection and microcephaly (Adibi et al., 2016; Gabriel et al., 2016). In this study, we investigated the molecular mechanisms that may be responsible for ZIKV-induced cell death during abnormal brain development. The genome sequences of ZIKV from the 2015 outbreak in humans provide us with a novel opportunity to predict the host factors that are required for infection by ZIKV (Ioos et al., 2014; Calvet et al., 2016; Cunha et al., 2016; Faria et al., 2016; Mlakar et al., 2016; Wang et al., 2016). It has been reported that the flaviviruses share a similar genomic organization and replication strategy, even though they cause a wide range of distinct clinical diseases in humans (Fernandez-Garcia et al., 2009). The viral proteins in the genus *Flavivirus* often interact with the same host proteins, so known flavivirus–host interactions may be useful in predicting ZIKV–human protein interactions (Huang et al., 2014; Wang et al., 2016). Furthermore, the protein similarities among the ZIKV-related viruses suggest that they interact with the same molecular factors in the host genome as does ZIKV. With the development of protein–protein interaction databases, computational approaches provide a viable alternative method of mapping genetic regulatory networks in the human genome (Pastorino et al., 2010; Shah et al., 2015).

In this study, a database of 248 ZIKV-related proteins in the human genome and 221 microcephaly-associated human proteins was created, and used to predict the molecular mechanisms involved in ZIKV infection and microcephaly. The results of this study indicate that these related proteins manipulate the cell death pathway through specific protein interactions during the genetic evolution of ZIKV, leading to a developmental disorder of the neurological system. Mechanisms by which ZIKV may be associated with microcephaly are presented here to provide a set of hypotheses for the further experimental investigation of the molecular mechanisms underlying ZIKV-induced cell death and to suggest targets for potential therapeutic drugs.

## Results

### P53 is the hub of the genetic regulatory network for ZIKV-related and microcephaly-associated proteins

**Figure 1:**
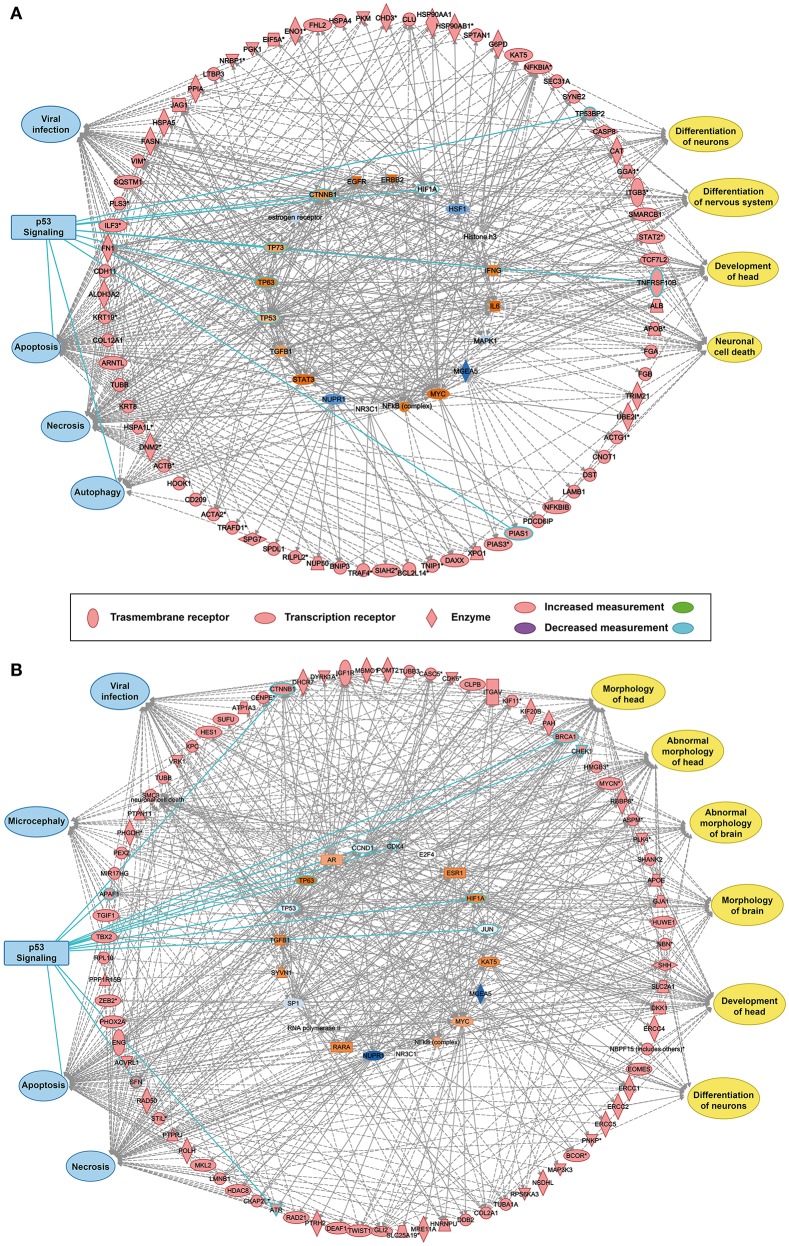
Transcriptional regulatory network and P53 signaling pathways associated with ZIKV infection and microcephaly. **(A,B)** Transcriptional regulatory networks of ZIKV-related **(A)** and microcephaly-associated **(B)** human proteins.

The reconstructed transcriptional regulatory network based on the predicted ZIKV-related and microcephaly-associated human proteins (Figures S1–S3) is shown in Figures 1A,B. The predicted upstream regulator of the ZIKV–human interaction proteins is related to brain development and brain diseases, such as neuronal cell death, the differentiation of neurons, the differentiation of the nervous system, and the development of the head (Figure 1A). The upstream regulators involved in viral infection clearly implicate the P53-dependent apoptotic pathway (Figure 1A), which interacts strongly with the transcriptional regulator in human cells to induce cell death in the brain. The reconstructed molecular regulatory network of the microcephaly-associated human proteins shows that the upstream regulatory factor is directly related to viral infection and microcephaly, which can damage the normal molecular regulation of the morphology of the head and brain, the development of head, and the differentiation of neurons, inducing an abnormal morphology of head (Figure 1B). Interestingly, the hub of this gene regulatory network is also the P53 protein (Figure 1B), which is the key co-regulator with P63, CCND1, CDK4, HIF1A, MDM2, and JUN in the P53 signaling pathway (Figures 1A,B).

### P53 cell death pathways play important roles in ZIKV infection and microcephaly

**Figure 2:**
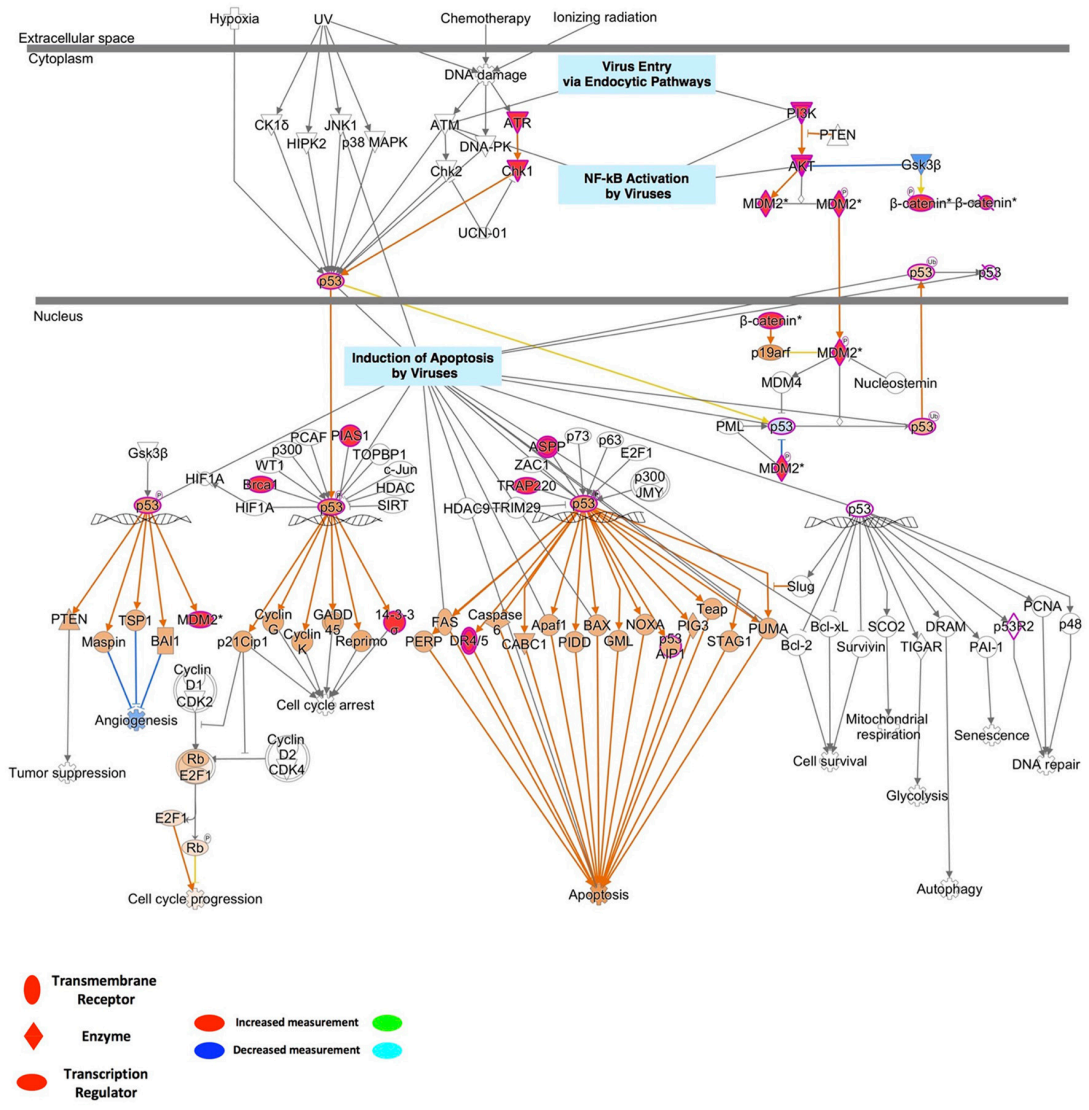
Detailed information on the key regulatory factors involved in P53 signaling that play important roles in the activation of apoptosis by viruses.

To identify the possible mechanistic functional pathway connecting the ZIKV-related and microcephaly-associated human proteins, pathway-related information was inferred with a phenotype annotation tool and used to perform functional and disease pathway enrichment analyses (Figures S4, S5). The diverse pathways relevant to ZVD include the mechanisms involved in the viral exit from the host cells, viral entry *via* endocytic pathways, and the activation of NF-κB by viruses. The death receptor signaling, apoptosis signaling, p53 signaling, and autophagy pathways are also implicated in the mechanisms of ZVD (Figure S4A). The microcephaly-associated human proteins are functionally closely related and are involved in multiple pathways, which are shared by the proteins involved in the ZIKV–human interaction. These pathways involve P53 signaling, NF-κB activation by viruses, the mechanisms of viral exit from the host cells, apoptosis signaling, and death receptor signaling (Figure S4B). These shared pathways are important in the human immune response to viral infection and cell apoptosis, especially the P53 signaling pathway. Notably, the finding that the ZIKV–human interaction proteins are involved in the death receptor signaling pathway implies that ZIKV infection induces brain disorders during fetal development *via* apoptosis or cell death, which are related to P53 signaling and the direct interaction of P53 with the MDM2 protein (Yang et al., 2008; Bhuvanakantham et al., 2010) in Figure 2. Figure 2 also provides detailed information about the key regulatory factors in the P53 signaling pathway that play important roles in the activation of apoptosis by viruses (Austin et al., 2012; Ghouzzi et al., 2016). These results suggest that ZIKV affects the MDM2-mediated P53 signaling pathway to induce programmed cell death, leading to defects in brain and neuron development.

### Interaction between ZCP and MDM2 is involved in P53-mediated apoptosis pathways

**Figure 3:**
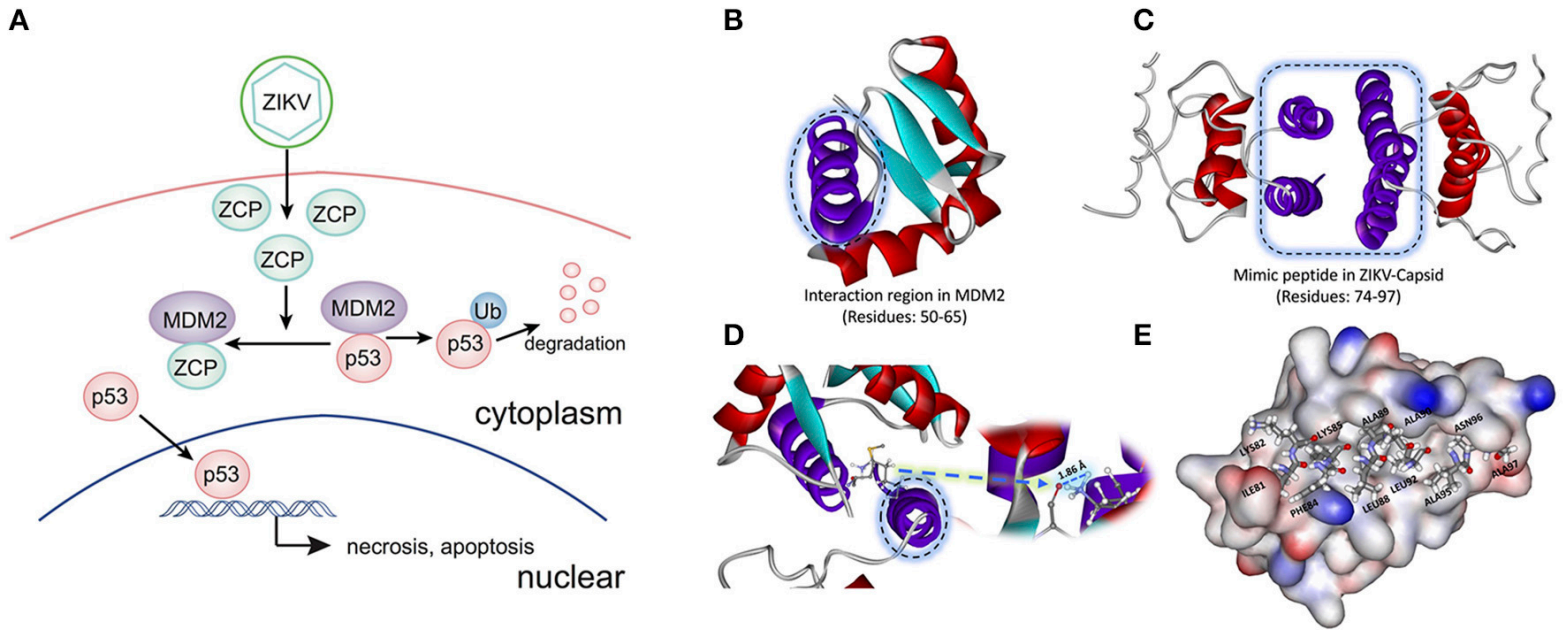
Schematic diagram and structure of the interaction between the ZIKV capsid protein (ZCP) and MDM2. **(A)** Schematic diagram of the interaction between ZCP and MDM2 in the P53-mediated apoptosis pathways. **(B)** Binding domain (residues 50–65) in the MDM2 structure (PDB ID: 3W69) that is critical for the P53–MDM2 interaction is shown as a purple helix. **(C)** Homology modeling of ZCP, in which the C-terminal helix is colored purple (residues 74–97). **(D)** Strong hydrogen bonding is shown at the O of MET50 (ball-and-stick model) in MDM2, which binds to HZ2 of LYS85 (ball-and-stick model) in ZCP. Right purple helix is the mimic ZCP peptide (residues 74–97). **(E)** Match between residues 81–97 (ILE81, LYS82, PHE84, LYS85, LEU88, ALA89, ALA91, LEU92, ALA95, ASN96, and ALA97) in the C-terminal regions of ZCP and MDM2. MDM2 protein is shown in the protein surface style and the residues of the mimic peptide are shown with sticks.

To determine the potential P53-induced cell death pathways regulated by MDM2 together with ZCP during ZVD, we used the strategy illustrated schematically in Figure 3A. The interaction region was identified as residues 50–65 (Figure 3B, purple helix; Figures S7A–C in Supplementary Materials) in the MDM2 structure (PDB ID: 3W69), in molecular docking and previous publications (Miyazaki et al., 2013). It is also the critical binding domain in the P53–MDM2 interaction and acts as a ubiquitin protein ligase, suppressing the transcriptional activity of P53 and promoting its degradation. This finding, based on previous bioinformatics data, suggests that the C-terminal helix of the ZCP (residues 74–97; Figure 3C, purple helix) mediates the effects of P53-induced cell death on brain development through a similar interaction with MDM2 (Yang et al., 2008; Bhuvanakantham et al., 2010).

Our molecular docking results indicate that the C-termini of ZCP and MDM2 form a strong hydrogen bond, in which the oxygen (O) of MET50 (ball-and-stick model) in MDM2 binds to the HZ2 of LYS85 (ball-and-stick model) in the C-terminal region of ZCP (Figure 3D and Figure S6). We also detected a hydrophobic interaction and steric matching between the C-termini of ZCP and MDM2. Residues 81–97 (ILE81, LYS82, PHE84, LYS85, LEU88, ALA89, ALA91, LEU92, ALA95, ASN96, and ALA97) at the C-terminus of ZCP matched the MDM2 protein tightly (Figure 3E). These hydrophobic interactions contribute to the interaction between the two proteins and may also play a key role in P53-mediated cell death during brain development.

**Figure 4:**
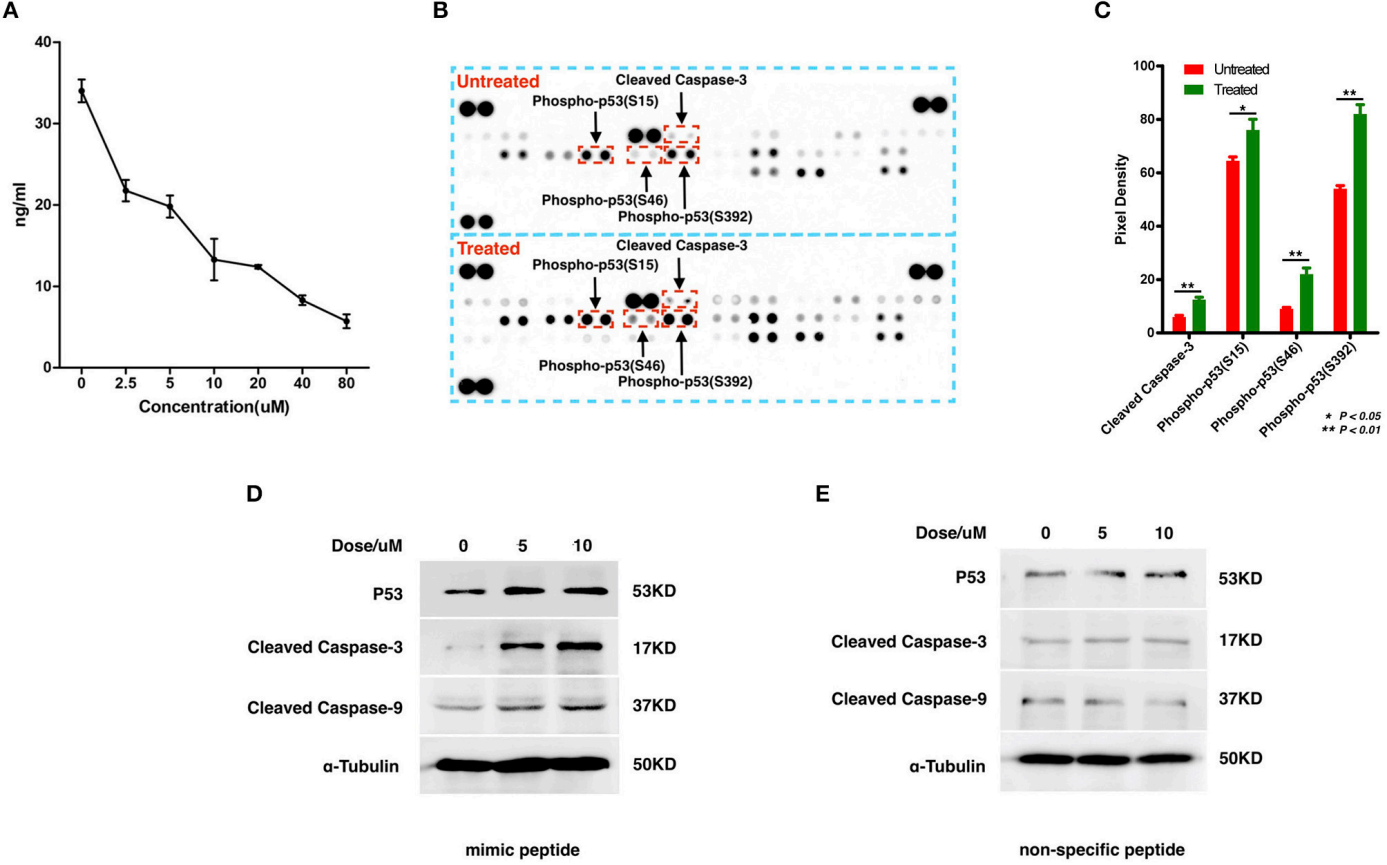
Detection of the interaction between MDM2 and the synthetic ZCP peptide during the activation of P53-induced cell death in U87 cells. **(A)** Competitive inhibition effect of treatment with the synthetic peptide on intracellular free MDM2 in U87 cells. The concentration of cellular free MDM2 was determined with an ELISA. Values shown are means ± SD. **(B)** P53 activation and apoptotic markers were upregulated in the synthetic-peptide-treated U87 cells compared with the control in the protein array. **(C)** Quantification of the markers in the images shown in **(B)**, signals were quantified with ImageJ. **(D)** Activation of total P53 and apoptotic markers were confirmed with western blotting. **(E)** Non-specific peptide. SD, standard deviation. ^*^P < 0.05; ^**^P < 0.01.

To examine the interaction between ZCP and MDM2, we synthesized a peptide with the C-terminal sequence of ZCP (mimic peptide; residues 74–97) to mimic ZCP. We used an enzyme-linked immunosorbent assay (ELISA) of human MDM2 to determine the concentration of free MDM2 in U87 cells after treatment with the synthetic peptide (Figure 4A). The results shown in Figure 4A indicate that the synthetic ZCP peptide reduced the cellular free MDM2 protein in the U87 cells, implying a probable direct interaction between ZCP and MDM2 *in vivo*. We then measured the activation of P53 and the cell death marker in U87 cells after treatment with the ZCP mimic (Figures 4B–D). This induced the activation of P53 and the apoptosis marker in the protein array (Figures 4B,C). We detected significantly increased levels of cleaved caspase 3 and the phosphorylation of Ser15, Ser46, and Ser392 in P53, which are well-established markers of P53 activation. The result of a western blotting analysis also showed increases in total P53, cleaved caspase 9, and cleaved caspase 3 proteins, which confirmed the activation of the P53-dependent apoptosis pathway (Figure 4D). We selected a non-specific peptide derived from ZCP (residues 30–53; Figure S7D) as the control peptide in subsequent experiments, because it does not interact with MDM2. The non-specific peptide did not affect the levels of those proteins in the treated cells. Consistent with our previous predictive results and recent reports, these results suggest that the synthetic peptide functionally mimics ZCP in the MDM2-mediated activation of P53 during the cell death process.

### Reduced cell viability and induction of apoptosis by mimic ZCP peptide in culture

**Figure 5:**
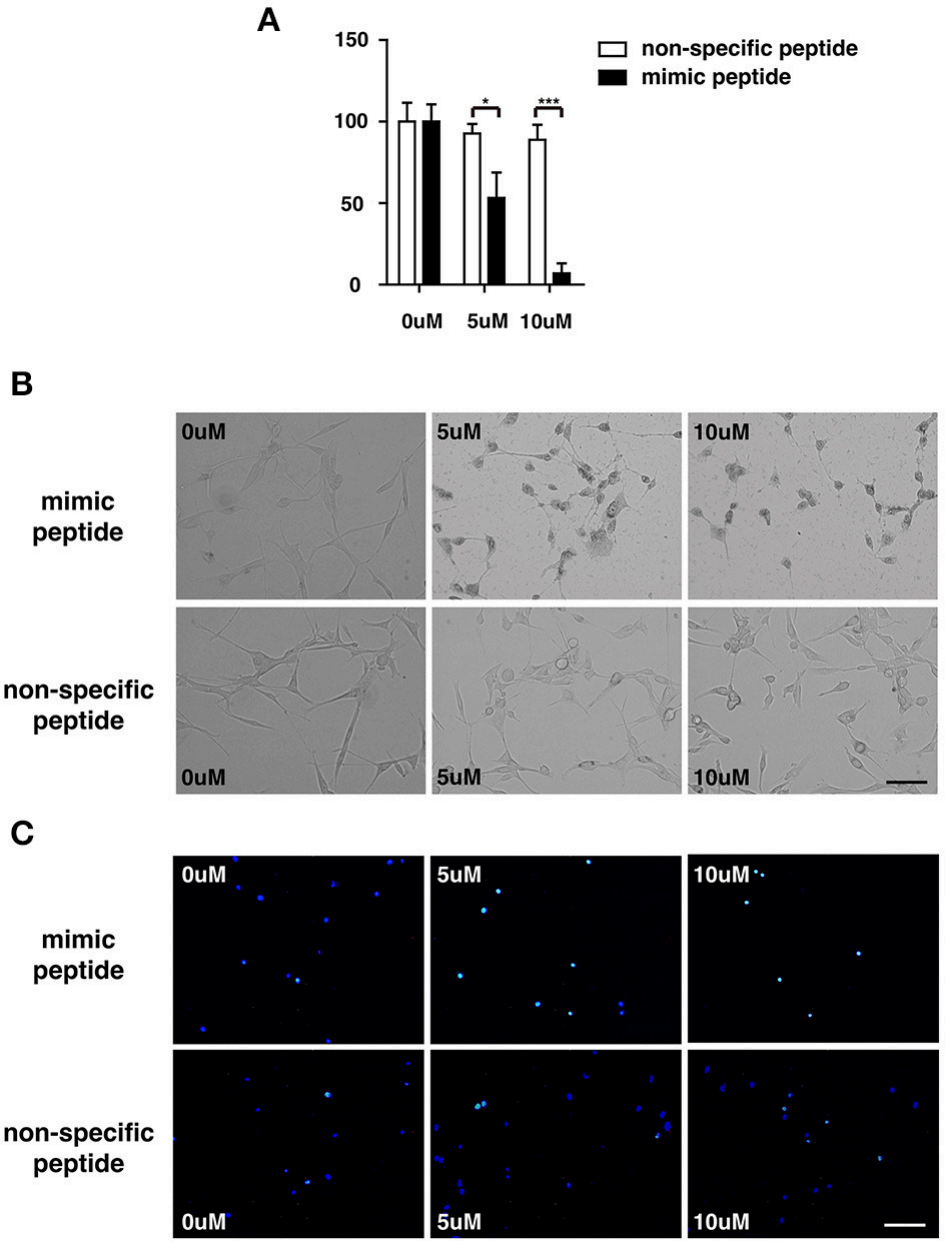
U87 cells were treated with a synthetic peptide derived from ZCP. **(A)** Effect of the synthetic peptide on the viability of U87 cells is shown. U87 cells were exposed to different concentrations (0, 5, or 10 μM) of the synthetic peptide for 4 h and cell viability was determined with the CCK-8 assay. Representative result is shown as the mean ± SD of triplicate wells from one of three independent experiments. **(B)** Morphology of U87 cells after incubation for 0, 6, or 12 h at 37°C with various peptide concentrations (0, 5, or 10 μM). **(C)** Fluorescent signals for TUNEL staining (green) and DAPI (blue) in U87 cells treated with 0, 5, or 10 μM ZCP peptide for 4 h at 37°C. Bars: 100 μm. SD, standard deviation. ^*^P < 0.05; ^**^P < 0.001.

To explore the potential involvement of ZCP in cell death *in vitro*, we first measured the viability of cultured U87 cells treated with the synthetic ZCP peptide, using a CCK-8 assay. The viability of the U87 cells decreased significantly and dose-dependently after treatment with the mimic peptide (Figure 5A). The U87 cells were then incubated with different concentrations (5 or 10 μM) of the synthetic peptide to mimic ZCP-induced cell death *in vitro*. Six hours after the addition of the synthetic ZCP peptide to the serum-free medium, a microscopic analysis revealed morphological changes typical of apoptosis in all the mimic-peptide-treated cells, but not in the non-specific-peptide-treated cells (Figure 5B). Significant necrosis and apoptotic bodies were also observed in the mimic-peptide-treated cells (Figure 5B). As shown in Figure 5C, all the mimic-peptide-treated U87 cells stained with TUNEL, and almost all of them stained positively in their nuclei. Apoptotic U87 cells displaying fluorescent signals were clearly evident after treatment for 6 h, when the cells were viewed with fluorescence microscopy. However, TUNEL staining remained at the baseline level in the cells treated with the non-specific peptide. This finding indicates that the mimic-peptide-treated cells were apoptotic.

### Synthetic ZCP mimic induces brain cell death *in vivo*

**Figure 6:**
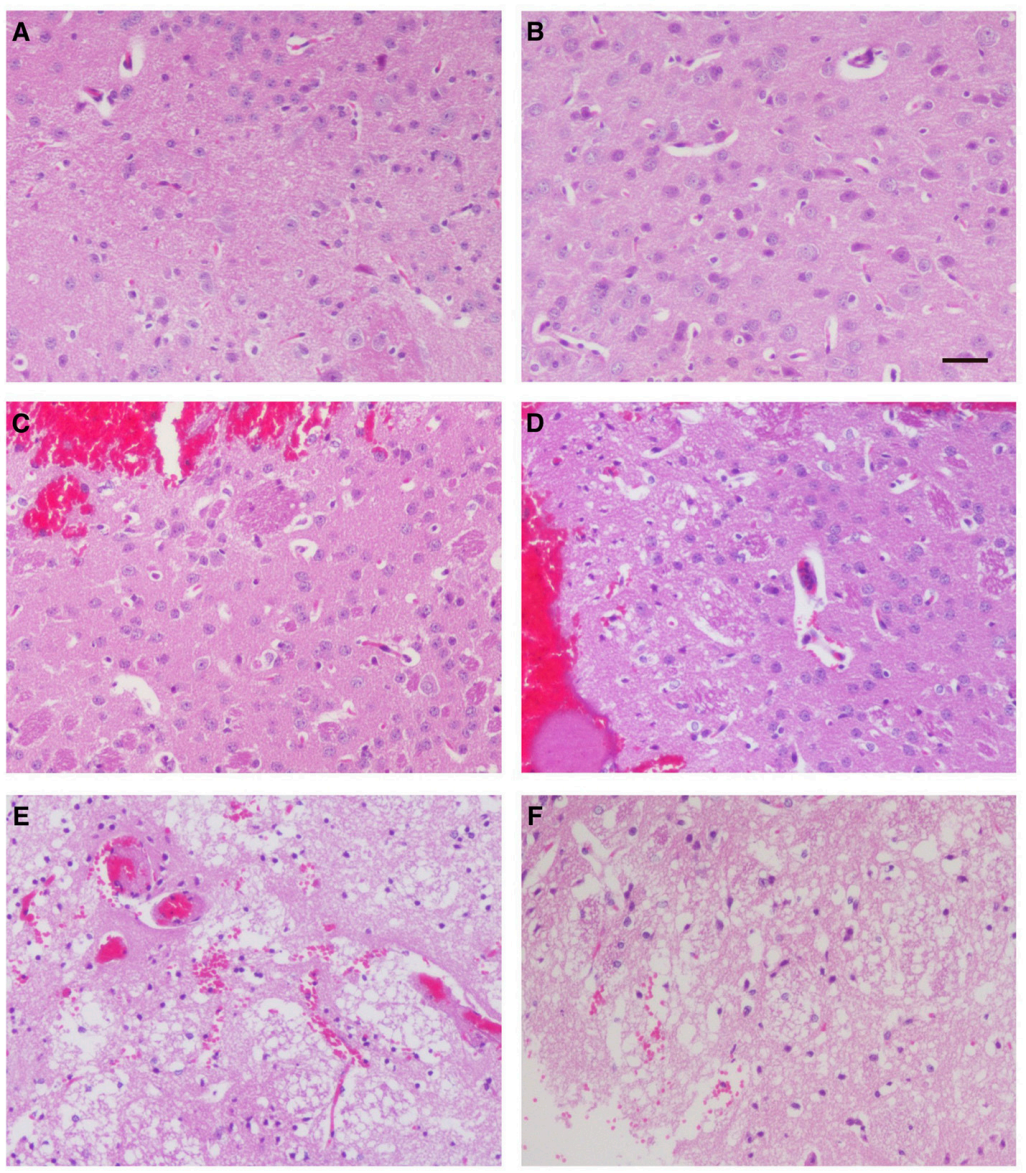
Histopathological examination of mouse brain tissues 6 h after injection. Hematoxylin-and-eosin staining of the caudate putamen in brain tissues from blank control mice **(A,B)**, control mice injected non-specific peptide **(C,D)**, and mice injected with the mimic ZCP peptide **(E,F)**. Bars: 100 μm.

To investigate whether ZCP induces brain injury *in vivo*, we injected the synthetic ZCP peptide directly into Balb/C mouse brains (Supplemental Videos). At 6 h after injection, the brains from the Group B and C mice were hypertrophic and displayed only mild swelling compared with those of Group A. In a histopathological analysis, hematoxylin and eosin (HE)-stained brain tissues revealed a small amount of degenerating or necrotic neuronal cells and the occasional infiltration of a few inflammatory cells in the caudate putamen in the brain sections near the injection point in the Group B mice (Figures 6C,D) compared with the Group A mice (Figures 6A,B). However, there were also large regions of light staining and obvious infiltration of inflammatory cells in the Group C mice (Figures 6E,F). HE staining and light microscopy also revealed massive necrosis in the brain tissues of the Group C mice, and the brain cells showed various types of degeneration, including cytolysis, nuclear fragmentation, and indistinct boundaries between the cells. Notably, exfoliated vascular endothelial cells were also observed (Figures 6E,F). The degree of brain tissue necrosis was significantly higher in the Group C mice than in the Group B mice, and the normal structure of the brain tissue was lost or slightly disordered (Figures 6E,F).

**Figure 7:**
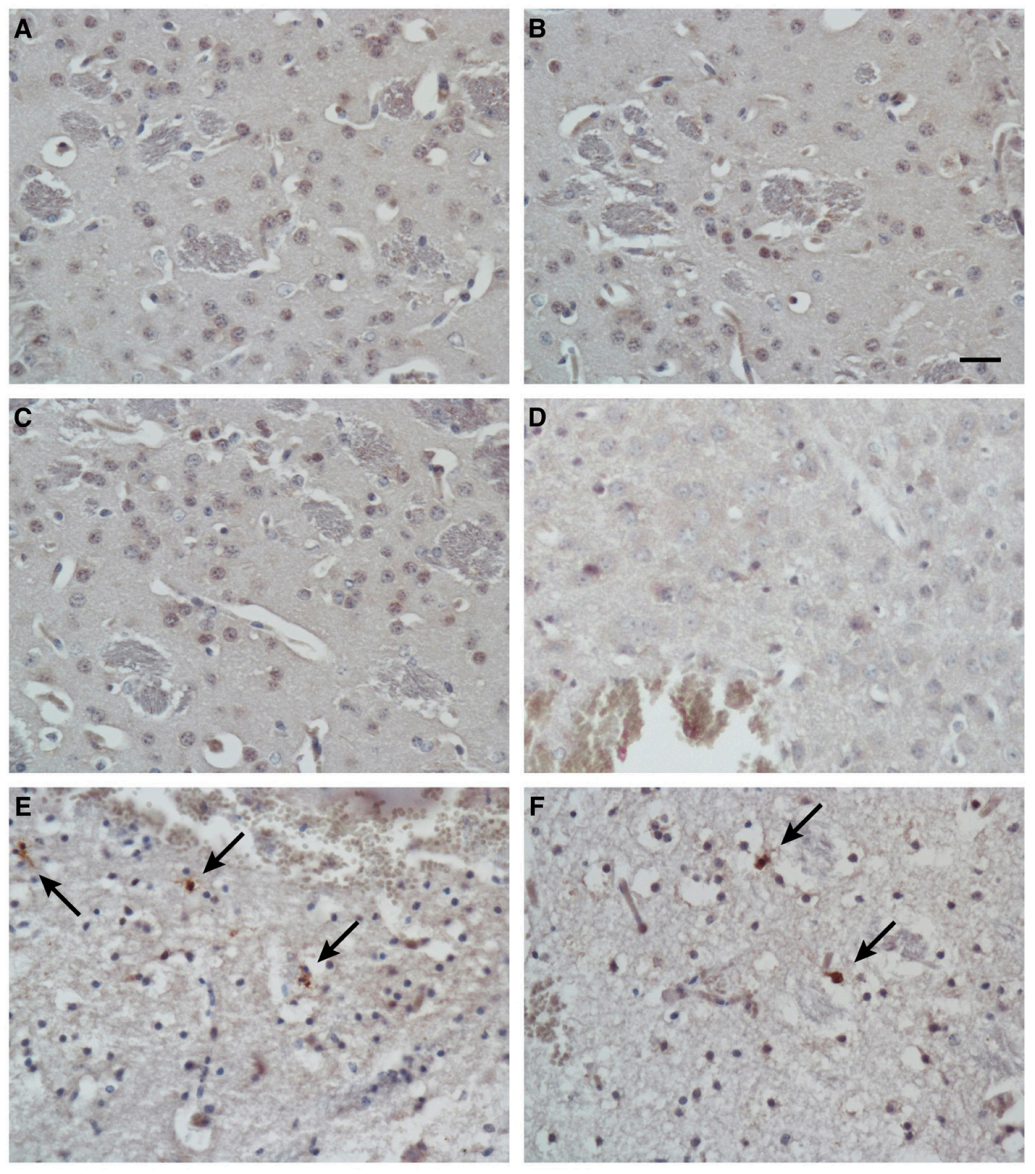
Immunohistochemical analysis of apoptotic cells in tissues after injection. Immunostaining for cleaved caspase 3 in brain tissues from blank control mice **(A,B)**, control mice injected with non-specific peptide **(C,D)**, and mice injected with synthetic ZCP mimic peptide **(E,F)**. Apoptosis increased dramatically in the synthetic-peptide-treated mouse brains. Arrow indicates cells positive for cleaved caspase 3. Bars: 100 μm.

To identify the apoptotic cells after treatment, the presence of cleaved caspase 3 in the tissues was detected with immunohistochemical techniques. In addition to the massive necrosis induced by the mimic peptide in the Group C mice, the number of apoptotic cells was increased, as revealed by immunostaining with an antibody directed against cleaved caspase 3 (Figures 7E,F). However, rare apoptotic cells were observed in the untreated mice (Group A; Figures 7A,B) and the non-specific-peptide-treated mice (Group B; Figures 7C,D).

## Discussion

ZIKV infection is an emerging threat around the world and appears to be associated with brain diseases such as microcephaly in infants (Calvet et al., 2016; Cauchemez et al., 2016; Mlakar et al., 2016; Rasmussen et al., 2016; Gabriel et al., 2017) and with Guillain–Barré syndrome (Brasil et al., 2016; Cao-Lormeau et al., 2016). A major concern associated with ZIKV infection is the apparent increased incidence of microcephaly in neonates born to ZIKV-infected mothers in Brazil (Calvet et al., 2016; Rubin et al., 2016). No specific antiviral medications or treatments are currently approved for ZVD because there has been little research into the molecular mechanisms underlying the potential association between ZIKV infection and brain diseases (Adibi et al., 2016; Bayer et al., 2016; Gabriel et al., 2016; Lazear et al., 2016; Nowakowski et al., 2016; Tang et al., 2016). Here, we created a possible genetic regulatory network for the proteins involved in the ZIKV–human interaction and microcephaly-associated proteins (Figures S1–S3 and Tables S1, S2). Systemic virology was used to generate this landscape map, which assumes that proteins within common signaling pathways are associated with the same upstream transcription factors (Figures 1A,B and Figure S4).

We found that the cell death signaling pathway is associated with both ZIKV infection and microcephaly and that P53 is the hub of the transcriptional regulatory network connecting ZIKV infection and microcephaly (Figure 2 and Figure S5). Therefore, we inferred that ZIKV-related proteins might overlap the microcephaly-associated proteins at the P53 signaling pathway, where they probably interact with the MDM2 protein, inducing viral-infection-induced cell death, thereby leading to a disorder characterized by abnormal brain development (Figure 3A). In accordance with the findings of earlier studies (Yang et al., 2008; Bhuvanakantham et al., 2010; Sips et al., 2012), the results of our molecular docking analysis suggest that the C-terminus of ZCP interacts with MDM2 *via* the hydrophobic interactions that contribute to the ZCP–MDM2 interactions (Figures 3B–E and Figure S6). The similarity between the ZCP–MDM2 interaction and the P53–MDM2 interaction suggests that ZCP blocks the formation of the MDM2–P53 complex, causing P53 release (Figure 4). When the MDM2-mediated degradation of P53 is prevented during brain development, the P53-mediated cell death pathway is activated, triggering the apoptosis of neural cells. To investigate whether ZCP is involved in cell death in the brain, we co-incubated a synthetic ZCP peptide with U87 cells (Figure 5) and injected them directly into the brains of Balb/C mice (Figures 6, 7 and Figure S8). The results of both *in vitro* and *in vivo* experiments demonstrated that the synthetic ZCP mimic induced the death of U87 cells and cells in the brains of mice. This was evident as changes in the morphological features of the cells and the presence of fluorescent staining characteristic of apoptosis, and the brain injury in the mice was also detected histopathologically. Understanding the molecular response to ZIKV infection will facilitate the discovery of clinically useful drugs for the treatment of ZVD and will accelerate the development of vaccines for the prevention of ZIKV infection (Kostyuchenko et al., 2016; Sirohi et al., 2016; Song et al., 2016).

## Materials and methods

### Constructing a genetic regulatory network

The integrated regulatory network was constructed based on a systematic integration of various high-throughput datasets. The transcriptional factors associated with the interaction genes were selected from the Transcriptional Regulatory Element Database (TRED). The integrated target genes–transcriptional factors regulatory network was constructed by using Cytoscape software v3.2.1 (cytoscape.org; Shannon et al., 2003), which is an open-source software for visualizing complex networks and integrating these networks with any type of attribute data.

### Systems analysis of gene ontology and biological pathways

To map each interaction protein into a known phenotype, we used the databases Online Mendelian Inheritance in Man (OMIM) and Genome-wide association studies (GWAS), which are comprehensive, authoritative compendiums of human genes and genetic phenotypes with their functional annotations and disease classifications. To assign enriched gene ontology (GO) terms for host factors, DAVID Bioinformatics Resources v6.7 (https://david.ncifcrf.gov/home.jsp) was used to query the list of identified host factors that were common to two or more studies. Molecular functions, biological processes, and cellular components that were identified with a confidence level of at least 95% were included in the analysis. For each topological module, a pathway-enrichment analysis of the biological pathways in which the module-related genes are believed to be involved was performed using DAVID online analytical tools. The pathway databases used in this study were KEGG (www.genome.jp/kegg), REACTOME (www.reactome.org), PANTHER (www.pantherdb.org), and BIOCARTA (www.biocarta.com) (Kanehisa et al., 2016; Mi et al., 2016).

### Molecular structure preparation and molecular docking

We used the ZIKV Capsid structure described in a previous publication (Ekins et al., 2016), which indicated that the modeled structure was reliable. We first performed a molecular dynamics (MD) simulation of the reported Capsid protein structures to be involved in the system composed of water and ions to obtain a stable three-dimensional structure of the ZIKV Capsid protein (see the detailed information in Data Sheet 1 as the Supplementary Materials). The system was equilibrated with an 80 ns MD simulation and the result of RMSD (Root-mean-square deviation) showed that the structure became convergent after 43 ns with a fluctuation range of 0.5–2.5 Å, probably during the 80 ns MD simulation (Figure S6). Next, we employed the stable structure of ZCP as the receptor to dock with the MDM2 protein. All molecular structure visualizations were performed with PyMOL (Version 1.7.2, http://pymol.org). In this study, AutoDock software version 4.2 (http://autodock.scripps.edu) (Morris et al., 2009) was employed to perform molecular docking as previously described for providing predictions of bound conformations. The ADT 1.5.6 (Auto Dock Tools) software was employed to prepare the docking procedures. The key residues for ZIKV Capsid structure were within the C-terminal of the Capsid. This region contains amino acid residues 65–97, and the number of runs was set to 100 in the molecular docking analysis. The MD simulation results were used to initiate the binding free energies calculations.

### Cell culture

U87 cells were grown at 37°C in a 5% CO_2_ atmosphere in Dulbecco's modified Eagle medium (DMEM) supplemented with 10% fetal bovine serum (Hyclone, USA). The medium was replaced every other day. Two synthetic peptide of the c-terminal sequence of ZIKV Capsid protein (Mimic peptide, residues 74–97, KKEAMEIIKKFKKDLAAMLRIINARRRRRRRR; Non-specific peptide, residues 30–53, LKRLPAGLLLGHGPIRMVLAILAFRRRRRRRR; Bankpeptide Co., Ltd., Hefei, China) was introduced to the U87 cells at a final concentration of 5 or 10 μM (ATCC, USA). The different experimental groups were cultured in six-well plates, with three replicates per group and each well contained 5 × 10^5^ cells. Cell morphological changes were observed by microscopy. Each experiment was repeated three times using cells from three different cell preparations.

### Cell viability

The Cell viability of U87 cells was determined using CCK-8 assay (Dojindo). U87 cells at the density of 1 × 10^4^ cells per well/100 μL were seeded in 96-well plate and cultured for 24 h to allow adherence and growth. After this time, cells were incubated with synthetic peptide using 0, 5, 10 μM doses for 4 h. After the treatment, cells were exposed to CCK-8 according to the manufacturer's instructions. The absorbance per well was measured at 540 nm using the POLARstar Omega Microplate Reader (BMG Labtech). Relative cell survival was expressed relative to the non-treated controls.

### Tunel assay

In Situ Cell Death Detection Kit, Fluorescein was obtained from Sigma-Aldrich. Staining was performed following the manufacturer's instruction. Cultured U87 cells were treated with mimic and non-specific peptide separately at the doses as labeled in figures. After 4 h peptide treatment, cells were fixed by 4% PFA Paraformaldehyde, PFA in PBS for 1 h at the room temperature. Cells were permeabilized using 0.1% Triton X-100 solution for 2 min on ice followed by twice rinsing by PBS. After the twice wash, cells were incubated with TUNEL reaction mixture for 1 h at 37°C in a humidified atmosphere in the dark. Cell nucleus was counterstained by DAPI. After PBS wash, the signal in cell nucleus was analyzed under fluorescent microscopy.

### Apoptosis array and immunoblotting

The effect of synthetic peptide to U87 cells was determined using the Human Apoptosis Array Kit from R&D Systems according to the manufacturer's instructions. Briefly, 2 × 10^6^ U87 cells per well/2 ML were seeded in 6-well plate 24 h before treatment. Cell lysate was harvested followed by treating with the synthetic peptide at the concentration of 0 and 10 μM for 2 h. Cell lysates were mixed with a cocktail of biotinylated detection antibodies. Incubated with the array membrane by the sample/antibody mixture. After a wash to remove unbound material, streptavidin-HRP and chemiluminescent detection reagents were added. Immunoblotting was performed according with standard protocols. The cells were lysed with 1% SDS lysis buffer, and the protein concentration was determined using a BCA Assay Kit (Pierce). Protein samples were resolved on SDS-PAGE and then were transferred to PVDF membranes. The membranes were blocked with 5% BSA and then were incubated with primary and secondary antibodies, and then washed. Protein expression was determined by using ECL Detection Reagent (Pierce). Antibodies against cleaved caspase-3, cleaved caspase-9 come from Cell Signaling Technology. Anti-α-tubulin was obtained from Sigma. Anti p53 was obtained from Santa Cruze.

### Elisa

MDM2 concentration was determined using human total MDM2/HDM2 DuoSet IC ELISA kit (R&D Systems) according to manufacturer's instructions. 2 × 10^6^ U87 cells per well/2 ML were seeded in 6-well plate. After the 24 h culture, cells were treated with the synthetic peptide at the concentration of 0, 2.5, 5, 10, 20, 40, 80 μM doses for 2 h. Cell lysates were harvested and incubated to the prepared 96-well assay plate. Detection antibody and diluted Streptaviding-HRP was added subsequently. The colorimetric substrate was added and developed. The absorbance value per well was determined at 540 nm using the POLARstar Omega Microplate Reader (BMG Labtech). A standard curve was generated from plotting the absorbance for each concentration of standard sample vs. the corresponding concentration. Sample otal protein concentration was determined by BCA assay (Pierce).

### Animals

Female BALB/C mice weighing 20–22 g were maintained for more than 1 week before commencing the experiment. The animals were housed in the animal facilities at Beijing Institute of Pharmacology and Toxicology. Animals received care according to the Division of Laboratory Animal Medicine guidelines, which were approved by the Association for Assessment and Accreditation of Laboratory Animal Care. This study was approved by the Committee on Animal Ethics in the Care and Use of Laboratory Animals of the Beijing Institute of Transfusion Medicine. All procedures were performed under general (Ethel) anesthesia using sterile surgical techniques.

### Injection studies and histopathologic analysis

Female BALB/C mice were randomly divided into three experimental groups with 10 mice in each group. Group A mice were used as a blank control; group B mice were injected with 5 μl of physiological saline containing 0.25 mg of non-specific peptide per mouse; group C mice were injected with 5 μl of physiologic saline containing 0.25 mg of the synthetic mimic peptide per mouse. After injection, all mice were monitored and housed in the animal facilities. Brain specimens were obtained at the specified time points and processed for histological examination. Paraffin-embedded sections (4 mm) were stained with HE. Paraffin-embedded sections (4 mm) were deparaffinized through graded ethanol solutions. After an antigen retrieval procedure of 30 min using target retrieval solution (DAKO), the sections were stained with the antibody of cleaved caspase-3 (Cell Signaling Technology) using the avidin-biotin complex system (Vector Laboratory). DAB was used as the substrate. Cell nuclei were counterstained with haematoxylin.

## Author contributions

YuT, XG, SfL, SxL, YJ., TH, BL, XA, and ZM characterized the materials and designed the experiments, under the supervision of DF and YiT, and PZ implemented the experiments under the supervision of DF and YuT wrote the manuscript with further contributions from and DF and YiT analyzed the data. All authors reviewed the manuscript.

### Conflict of interest statement

The authors declare that the research was conducted in the absence of any commercial or financial relationships that could be construed as a potential conflict of interest.

## Supplementary material

Figure S1 Reconstructed ZIKV–human interaction protein network, including 248 human proteins that potentially interact with ZIKV proteins.

Figure S2 The 212 proteins predicted to be associated with microcephaly that map to the interaction network.

Figure S3 Functional and disease analyses of the interaction network composed of ZIKV-related and microcephaly-associated proteins. **(A)** Functional mapping of ZIKV-related proteins. **(B)** Functional mapping of microcephaly-associated proteins.

Figure S4 Gene ontology (GO) enrichment analysis of the ZIKV-infection-associated and microcephaly-associated proteins. The ZIKV–human interaction proteins **(A–C)** and microcephaly-associated proteins **(D–F)** identified were classified using the GO and DAVID databases, which are based on molecular functions **(A,D)**, biological processes **(B,E)**, and cellular compartments **(C,F)**.

Figure S5 Bioinformatics analysis of the signaling pathways enriched during ZIKV infection or microcephaly. **(A,B)** Pathways most enriched during ZIKV infection **(A)** or microcephaly **(B)**. **(C)** Proteins involved in the ZIKV–human interaction are also involved in the death receptor signaling pathway, with detailed information on how the components relate to brain disorders during fetal development, *via* apoptosis or cell death.

Figure S6 RMSD–time curves for the ZIKV capsid protein (ZCP) in the 80 ns MD simulation indicated that the structure of ZCP was in a stable state.

Figure S7 Tetramer of ZCP (residues 25–104); ZPC mimic peptide (residues 74–104) is a purple helix; non-specific ZCP peptide (residues 30–53) is a green helix **(A)**. The docking complex of MDM2 and the ZCP peptide (residues 81–104; green helix). No interaction with MDM2 at residues 83, 99, 100, and 103 in the ZCP peptide **(B)**. Monomer of ZCP (residues 25–104); ZCP mimic peptide (residues 74–104; purple helix) and non-specific ZCP peptide (residues 30–53; green helix) **(C)**. Docking complex of MDM2 and the non-specific ZCP peptide (residues 30–53; green helix); there was no region of interaction between them **(D)**.

Figure S8 Histopathological examination of mouse brain tissues at 6 h after injection. Hematoxylin and eosin staining of brain tissues from blank control mice **(A,B)**, control mice injected non-specific peptide **(C,D)** and mice injected with mimic peptide of the ZCP protein **(E,F)**. Bars: 100 μm.

Table S1 ZIKV-related proteins in the human genome.

Table S2 Microcephaly-associated proteins in the human genome.
