# Supplementary figures and images for: An Integrative Analysis Reveals a Central Role of P53 Activation via MDM2 in Zika Virus Infection Induced Cell Death
Source: Front Cell Infect Microbiol. 2017 Jul 20;7:327. doi: 10.3389/fcimb.2017.00327 (PMC5517408; doi:10.3389/fcimb.2017.00327)

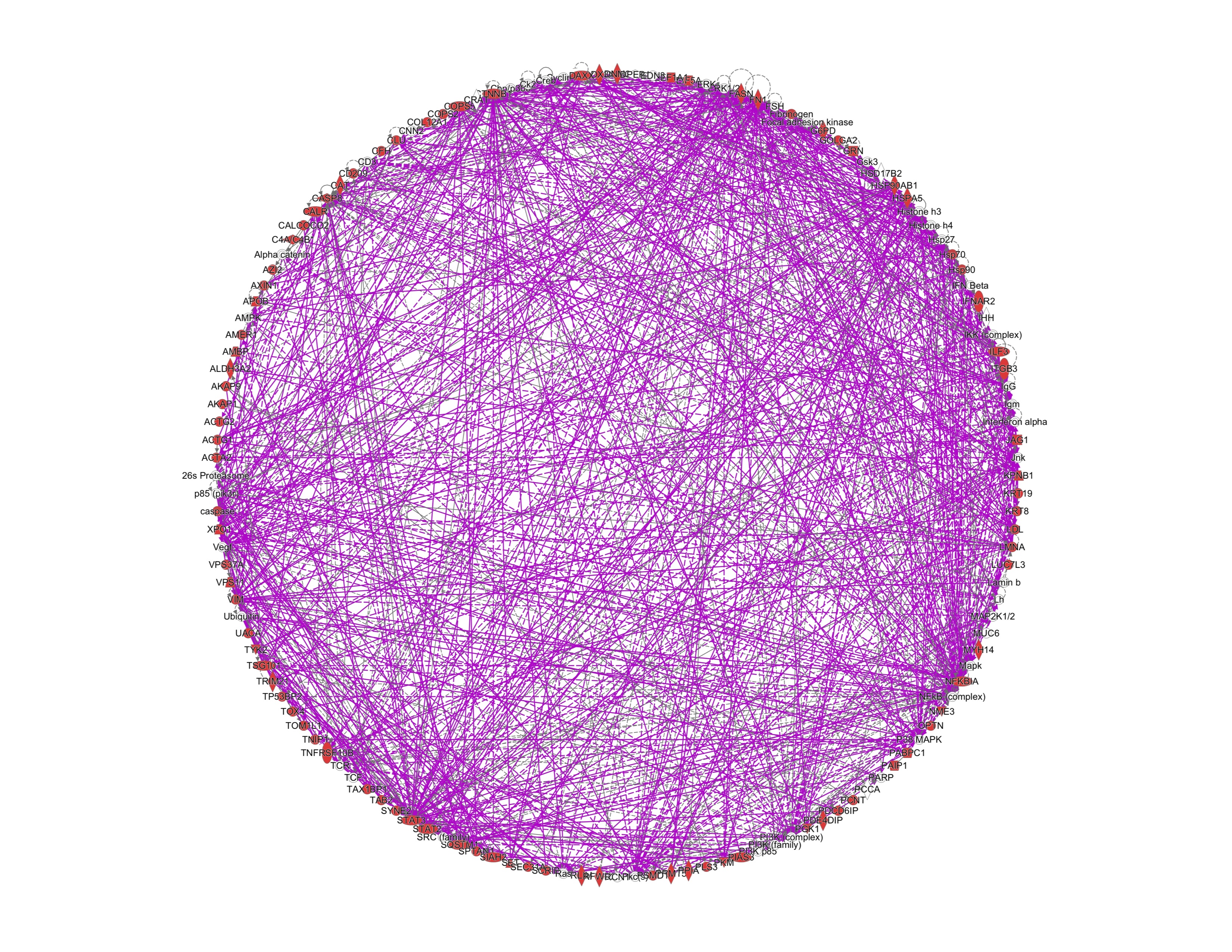

Supplement: Figure S1 — Reconstructed ZIKV–human interaction protein network, including 248 human proteins that potentially interact with ZIKV proteins. [file Image1.JPEG]

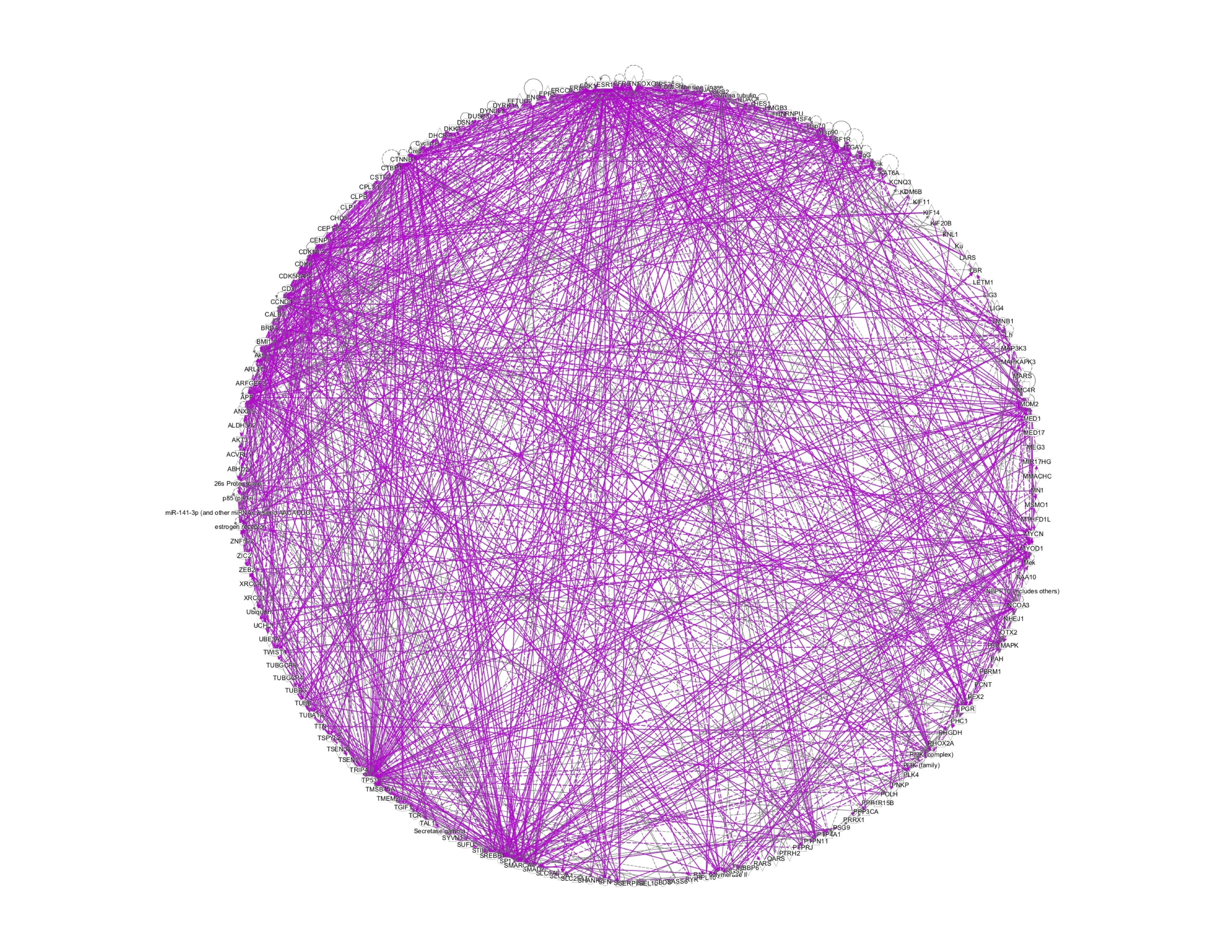

Supplement: Figure S2 — The 212 proteins predicted to be associated with microcephaly that map to the interaction network. [file Image2.JPEG]

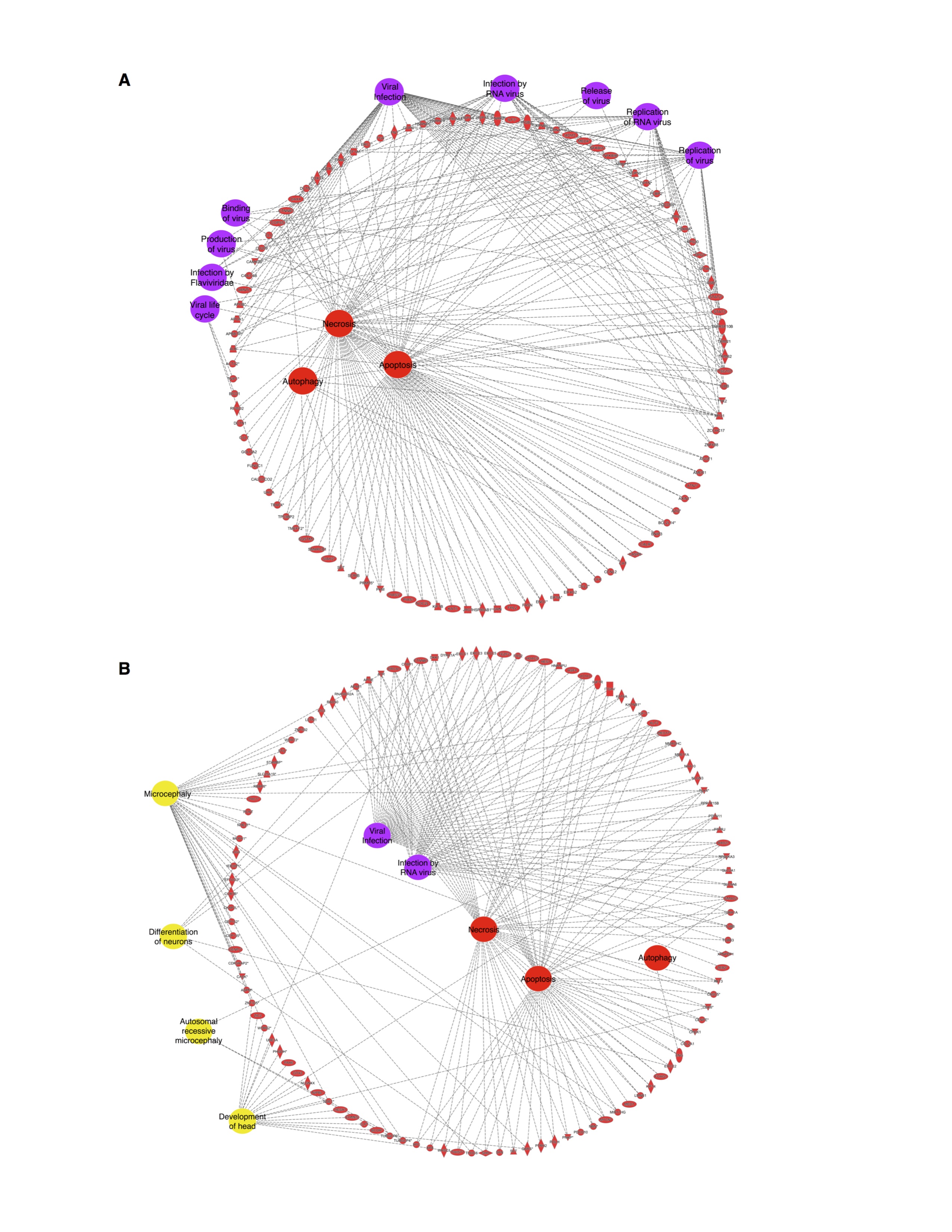

Supplement: Figure S3 — Functional and disease analyses of the interaction network composed of ZIKV-related and microcephaly-associated proteins. (A) Functional mapping of ZIKV-related proteins. (B) Functional mapping of microcephaly-associated proteins. [file Image3.JPEG]

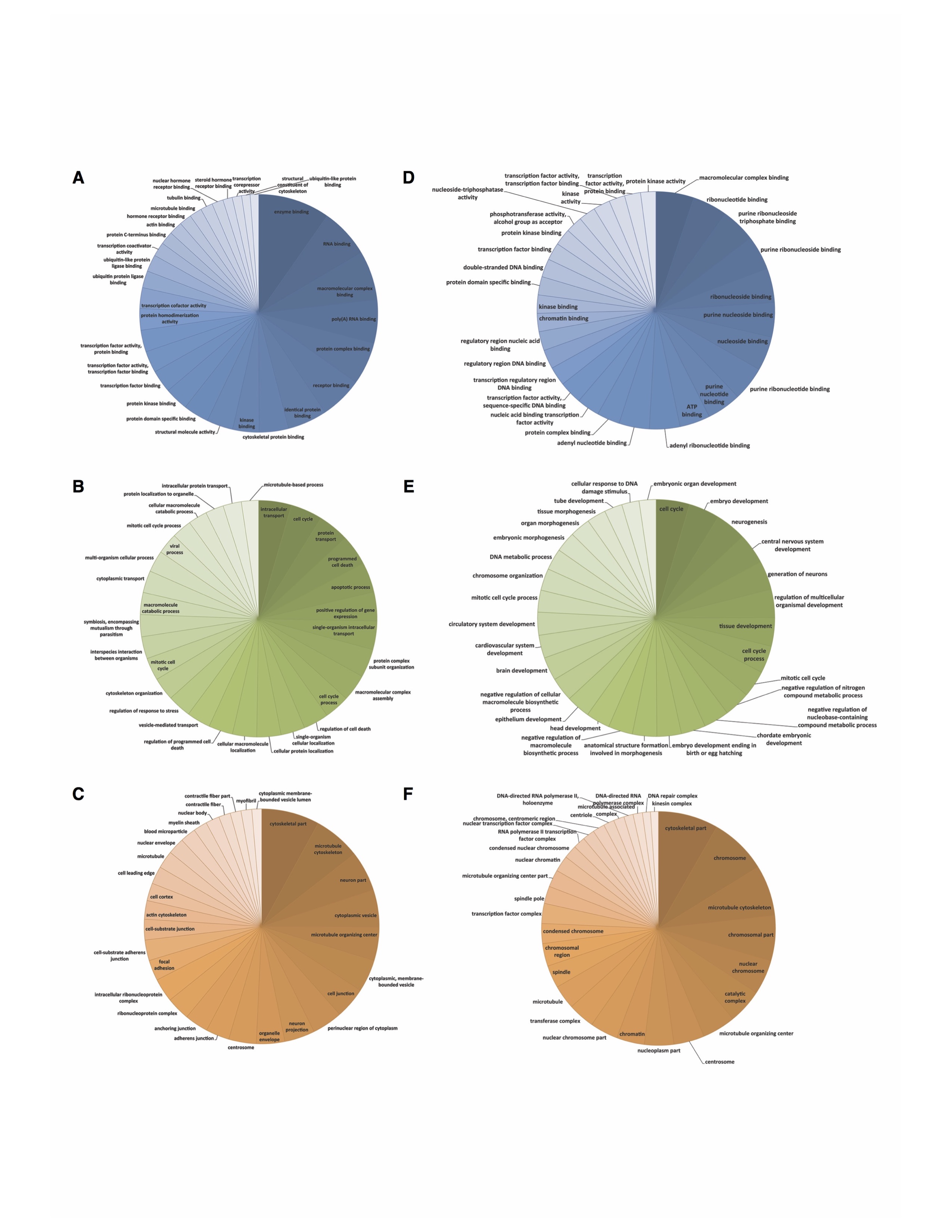

Supplement: Figure S4 — Gene ontology (GO) enrichment analysis of the ZIKV-infection-associated and microcephaly-associated proteins. The ZIKV–human interaction proteins (A–C) and microcephaly-associated proteins (D–F) identified were classified using the GO and DAVID databases, which are based on molecular functions (A,D), biological processes (B,E), and cellular compartments (C,F). [file Image4.JPEG]

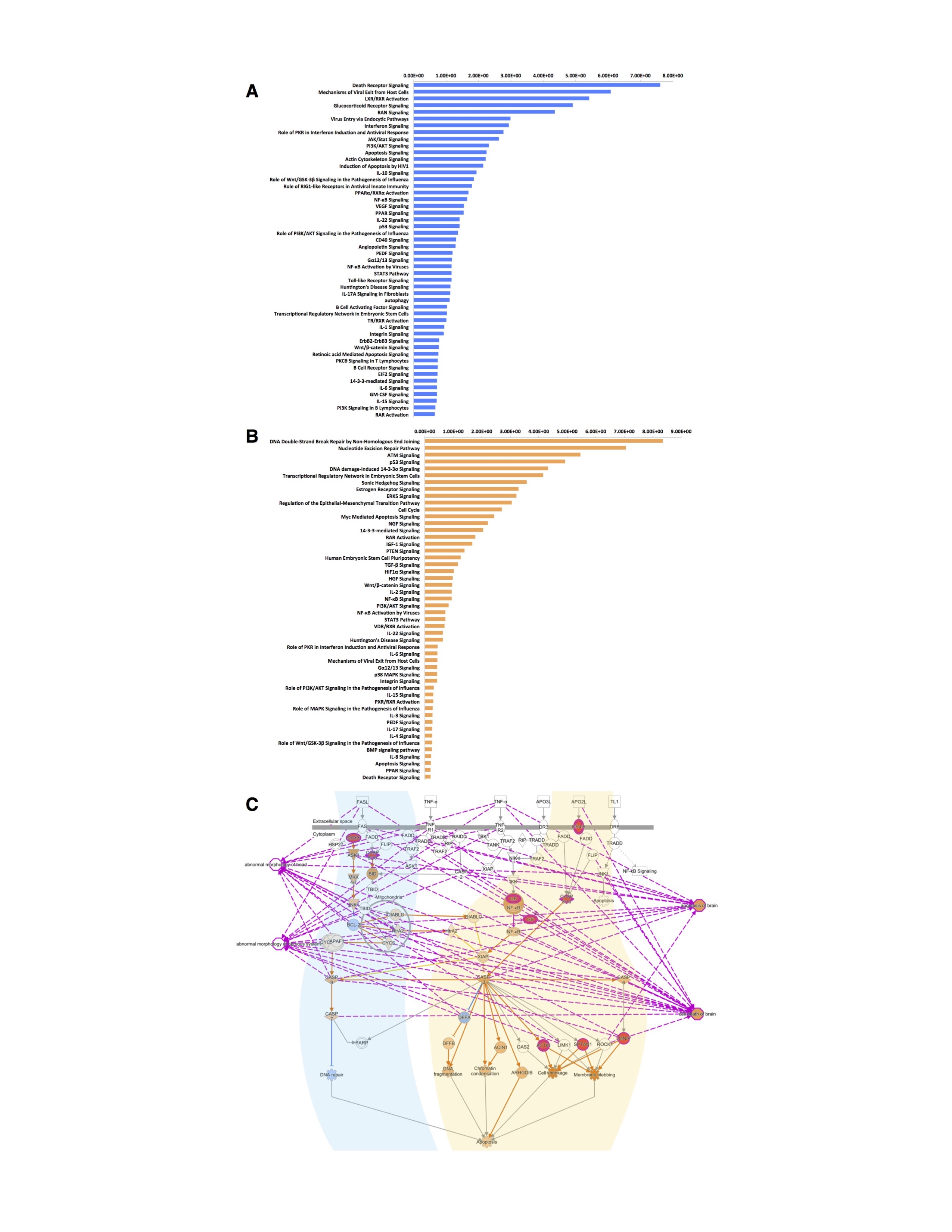

Supplement: Figure S5 — Bioinformatics analysis of the signaling pathways enriched during ZIKV infection or microcephaly. (A,B) Pathways most enriched during ZIKV infection (A) or microcephaly (B). (C) Proteins involved in the ZIKV–human interaction are also involved in the death receptor signaling pathway, with detailed information on how the components relate to brain disorders during fetal development, via apoptosis or cell death. [file Image5.JPEG]

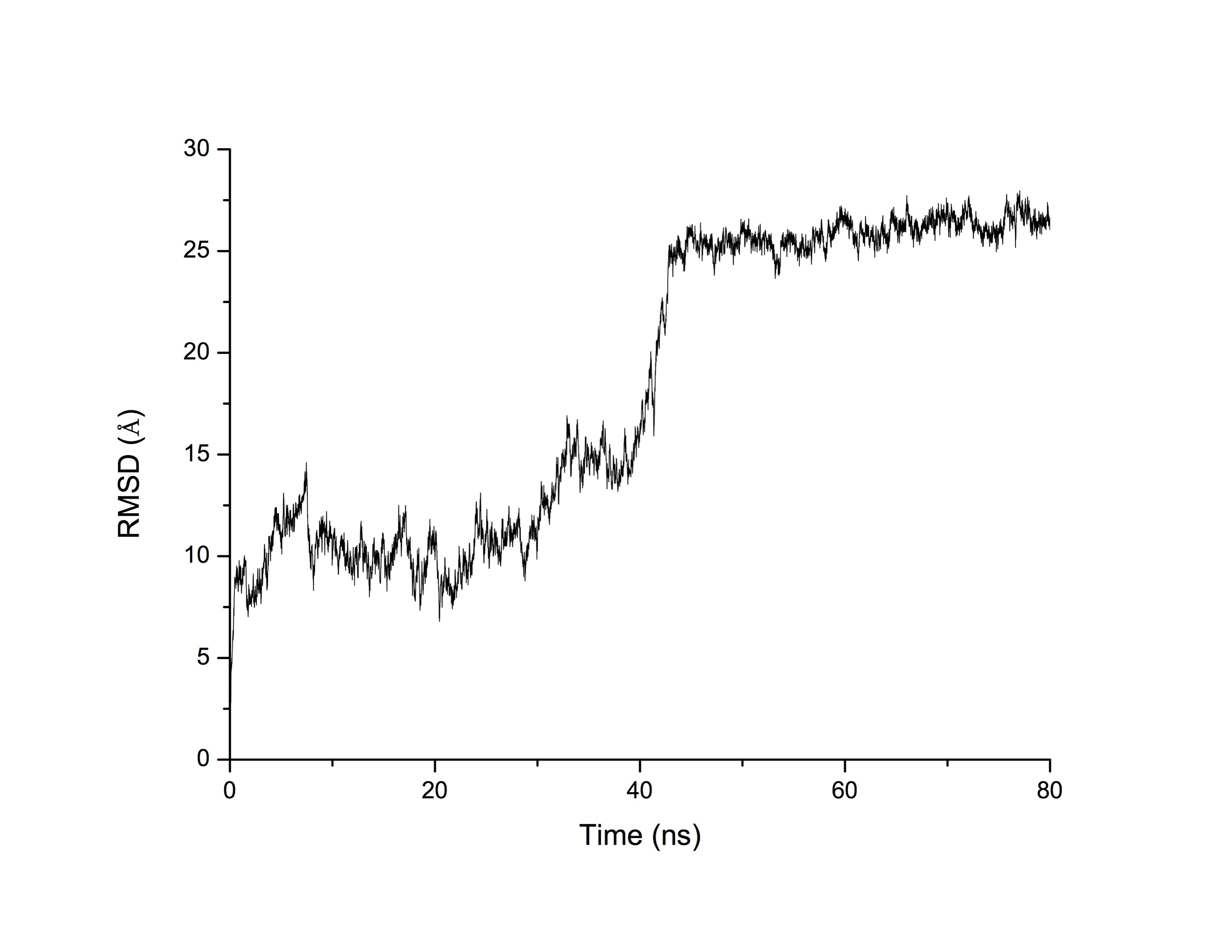

Supplement: Figure S6 — RMSD–time curves for the ZIKV capsid protein (ZCP) in the 80 ns MD simulation indicated that the structure of ZCP was in a stable state. [file Image6.JPEG]

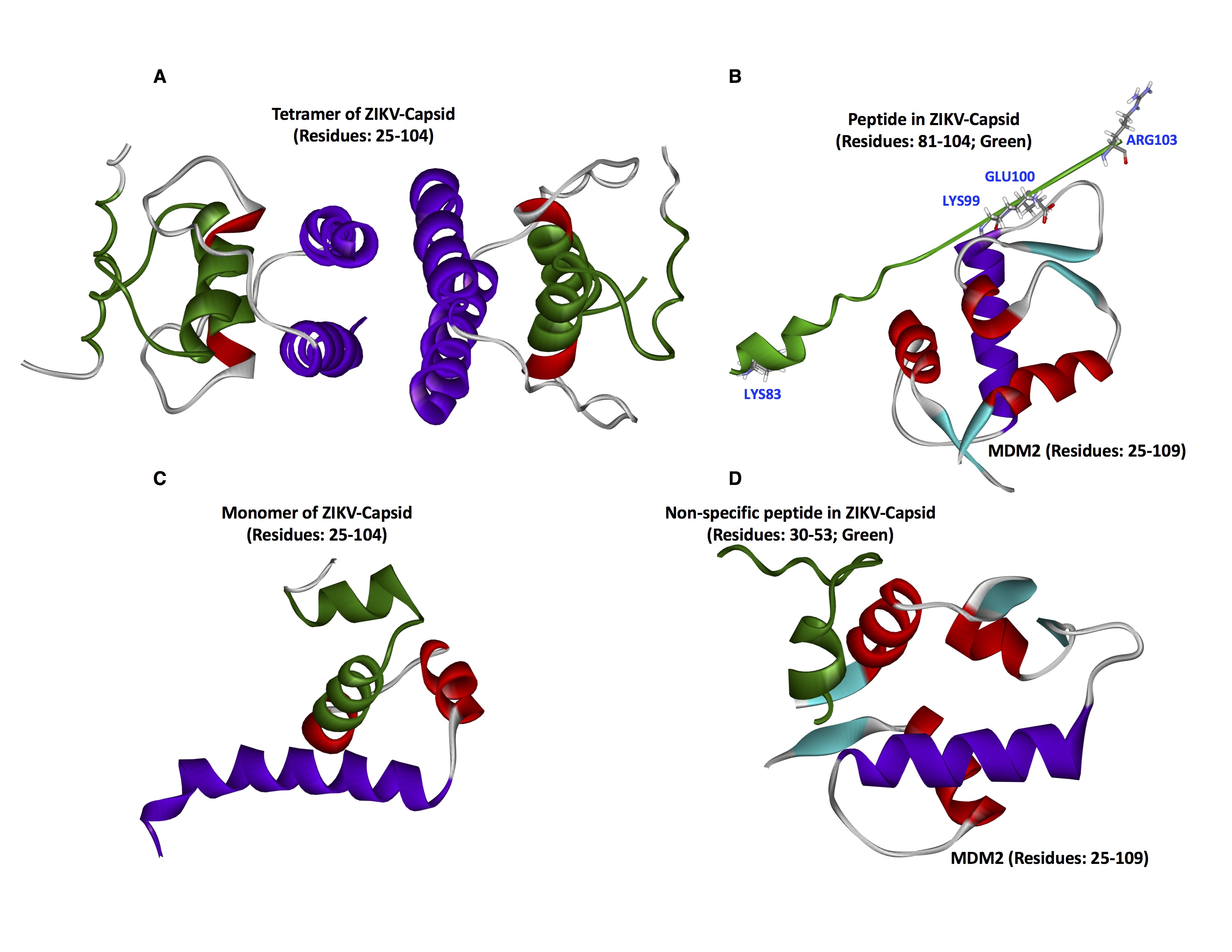

Supplement: Figure S7 — Tetramer of ZCP (residues 25–104); ZPC mimic peptide (residues 74–104) is a purple helix; non-specific ZCP peptide (residues 30–53) is a green helix (A). The docking complex of MDM2 and the ZCP peptide (residues 81–104; green helix). No interaction with MDM2 at residues 83, 99, 100, and 103 in the ZCP peptide (B). Monomer of ZCP (residues 25–104); ZCP mimic peptide (residues 74–104; purple helix) and non-specific ZCP peptide (residues 30–53; green helix) (C). Docking complex of MDM2 and the non-specific ZCP peptide (residues 30–53; green helix); there was no region of interaction between them (D). [file Image7.JPEG]
