# Supplementary material for: An Integrative Analysis Reveals a Central Role of P53 Activation via MDM2 in Zika Virus Infection Induced Cell Death
Source: Front Cell Infect Microbiol. 2017 Jul 20;7:327. doi: 10.3389/fcimb.2017.00327 (PMC5517408; doi:10.3389/fcimb.2017.00327)
Supplement: Table S1 — ZIKV-related proteins in the human genome. [file Table1.PDF]

Table S1 ZIKA-human interaction proteins

Human Symbol	Gene ID	Entrez Gene Name	Location	Type(s)
ACTA2	59	actin, alpha 2, smooth muscle, aorta	Cytoplasm	other
ACTB	60	actin, beta	Cytoplasm	other
ACTG1	71	actin gamma 1	Cytoplasm	other
ACTG2	72	actin, gamma 2, smooth muscle, enteric	Cytoplasm	other
AGGF1	55109	angiogenic factor with G patch and FHA domains 1	Cytoplasm	other
AKAP1	8165	A kinase (PRKA) anchor protein 1	Cytoplasm	other
AKAP8L	26993	A kinase (PRKA) anchor protein 8-like	Nucleus	other
AKAP9	10142	A kinase (PRKA) anchor protein 9	Cytoplasm	other
AKR7A3	22977	aldo-keto reductase family 7, member A3 (aflatoxin aldehyde reductase)	Cytoplasm	enzyme
ALB	213	albumin	Extracellular Space	transporter
ALDH3A2	224	aldehyde dehydrogenase 3 family, member A2	Cytoplasm	enzyme
AMBP	259	alpha-1-microglobulin/bikunin precursor	Extracellular Space	transporter
AMER1	139285	APC membrane recruitment protein 1	Plasma Membrane	other
ANKRD50	57182	ankyrin repeat domain 50	Other	other
APBB1IP	54518	amyloid beta (A4) precursor protein-binding, family B, member 1 interacting protein	Cytoplasm	other
APOA1	335	apolipoprotein A-I	Extracellular Space	transporter
APOB	338	apolipoprotein B	Extracellular Space	transporter
ARHGAP25	9938	Rho GTPase activating protein 25	Cytoplasm	other
ARID2	196528	AT rich interactive domain 2 (ARID, RFX-like)	Nucleus	transcription regulator
ARNTL	406	aryl hydrocarbon receptor nuclear translocator-like	Nucleus	transcription regulator
ATN1	1822	atrophin 1	Nucleus	transcription regulator
ATP6V1E1	529	ATPase, H+ transporting, lysosomal 31kDa, V1 subunit E1	Cytoplasm	transporter
AXIN1	8312	axin 1	Cytoplasm	other
AZI2	64343	5-azacytidine induced 2	Cytoplasm	other
BCL2L14	79370	BCL2-like 14 (apoptosis facilitator)	Cytoplasm	other
BICD1	636	bicaudal D homolog 1 (Drosophila)	Cytoplasm	other
BMS1	9790	BMS1 ribosome biogenesis factor	Nucleus	other
BNIP3	664	BCL2/adenovirus E1B 19kDa	Cytoplasm	other

		interacting protein 3		
C4A	720	complement component 4A (Chido blood group)	Extracellular Space	other
C4B	721	complement component 4B (Chido blood group)	Extracellular Space	other
CALCOCO2	10241	calcium binding and coiled-coil domain 2	Nucleus	other
CALR	811	calreticulin	Cytoplasm	transcription regulator
CAMK2B	816	calcium/calmodulin-dependent protein kinase II beta	Cytoplasm	kinase
CAMTA2	23125	calmodulin binding transcription activator 2	Nucleus	other
CASP8	841	caspase 8, apoptosis-related cysteine peptidase	Nucleus	peptidase
CAT	847	catalase	Cytoplasm	enzyme
CCDC14	64770	coiled-coil domain containing 14	Cytoplasm	other
CCDC66	285331	coiled-coil domain containing 66	Other	other
CCDC88C	440193	coiled-coil domain containing 88C	Cytoplasm	other
CCNL2	81669	cyclin L2	Nucleus	other
CD209	30835	CD209 molecule	Plasma Membrane	other
CDH11	1009	cadherin 11, type 2, OB-cadherin (osteoblast)	Plasma Membrane	other
CEP135	9662	centrosomal protein 135kDa	Cytoplasm	other
CEP250	11190	centrosomal protein 250kDa	Nucleus	other
CEP290	80184	centrosomal protein 290kDa	Nucleus	other
CEP63	80254	centrosomal protein 63kDa	Cytoplasm	other
CFAP157	286207	cilia and flagella associated protein 157	Other	other
CFH	3075	complement factor H	Extracellular Space	other
CGNL1	84952	cingulin-like 1	Plasma Membrane	other
CHD3	1107	chromodomain helicase DNA binding protein 3	Nucleus	enzyme
CLIP1	6249	CAP-GLY domain containing linker protein 1	Cytoplasm	other
CLU	1191	clusterin	Cytoplasm	other
CNOT1	23019	CCR4-NOT transcription complex, subunit 1	Cytoplasm	other
COBLL1	22837	cordon-bleu WH2 repeat protein-like 1	Extracellular Space	other
COL12A1	1303	collagen, type XII, alpha 1	Extracellular Space	other
COPS2	9318	COP9 signalosome subunit 2	Cytoplasm	other
COPS5	10987	COP9 signalosome subunit 5	Nucleus	transcription

				regulator
CTNNB1	1499	catenin (cadherin-associated protein), beta 1, 88kDa	Nucleus	transcription regulator
CUL7	9820	cullin 7	Cytoplasm	other
DAXX	1616	death-domain associated protein	Nucleus	transcription regulator
DCTN1	1639	dynactin 1	Cytoplasm	other
DCTN2	10540	dynactin 2 (p50)	Cytoplasm	other
DCUN1D4	23142	DCN1, defective in cullin neddylation 1, domain containing 4	Nucleus	other
DDX11L8	100302090	DEAD/H (Asp-Glu-Ala-Asp/His) box helicase 11 like 8	Other	other
DDX3X	1654	DEAD (Asp-Glu-Ala-Asp) box helicase 3, X-linked	Cytoplasm	enzyme
DDX5	1655	DEAD (Asp-Glu-Ala-Asp) box helicase 5	Nucleus	enzyme
DNM2	1785	dynamin 2	Plasma Membrane	enzyme
DNTTIP2	30836	deoxynucleotidyltransferase, terminal, interacting protein 2	Nucleus	other
DSCR3	10311	Down syndrome critical region 3	Nucleus	other
DST	667	dystonin	Plasma Membrane	other
DVL2	1856	dishevelled segment polarity protein 2	Cytoplasm	other
DYX1C1	161582	dyslexia susceptibility 1 candidate 1	Nucleus	other
EBNA1BP2	10969	EBNA1 binding protein 2	Nucleus	other
ECI2	10455	enoyl-CoA delta isomerase 2	Cytoplasm	enzyme
EEF1A1	1915	eukaryotic translation elongation factor 1 alpha 1	Cytoplasm	translation regulator
EID1	23741	EP300 interacting inhibitor of differentiation 1	Nucleus	transcription regulator
EIF4G2	1982	eukaryotic translation initiation factor 4 gamma, 2	Cytoplasm	translation regulator
EIF5A	1984	eukaryotic translation initiation factor 5A	Cytoplasm	translation regulator
EMILIN1	11117	elastin microfibril interfacer 1	Extracellular Space	other
ENO1	2023	enolase 1, (alpha)	Cytoplasm	enzyme
ENOX2	10495	ecto-NOX disulfide-thiol exchanger 2	Plasma Membrane	enzyme
ERC1	23085	ELKS/RAB6-interacting/CAST family member 1	Cytoplasm	other
FAM184A	79632	family with sequence similarity 184, member A	Extracellular Space	other
FAM92B	339145	family with sequence similarity 92, member B	Other	other
FASN	2194	fatty acid synthase	Cytoplasm	enzyme

FGA	2243	fibrinogen alpha chain	Extracellular Space	other
FGB	2244	fibrinogen beta chain	Extracellular Space	other
FHL2	2274	four and a half LIM domains 2	Nucleus	transcription regulator
FN1	2335	fibronectin 1	Extracellular Space	enzyme
FUNDC1	139341	FUN14 domain containing 1	Cytoplasm	other
FUNDC2	65991	FUN14 domain containing 2	Cytoplasm	other
FYB	2533	FYN binding protein	Nucleus	other
G6PD	2539	glucose-6-phosphate dehydrogenase	Cytoplasm	enzyme
GC	2638	group-specific component (vitamin D binding protein)	Extracellular Space	transporter
GDI2	2665	GDP dissociation inhibitor 2	Cytoplasm	other
GGA1	26088	golgi-associated, gamma adaptin ear containing, ARF binding protein 1	Cytoplasm	transporter
GOLGA2	2801	golgin A2	Cytoplasm	other
GOLGA6L9	440295	golgin A6 family-like 9	Other	other
GOLGA8J	653073	golgin A8 family, member R	Other	other
GOLGB1	2804	golgin B1	Cytoplasm	other
GOPC	57120	golgi-associated PDZ and coiled-coil motif containing	Cytoplasm	transporter
GPATCH2L	55668	G patch domain containing 2-like	Other	other
GRN	2896	granulin	Extracellular Space	growth factor
HAUS4	54930	HAUS augmin-like complex, subunit 4	Cytoplasm	other
HECW1	23072	HECT, C2 and WW domain containing E3 ubiquitin protein ligase 1	Cytoplasm	enzyme
HNRNPC	3183	heterogeneous nuclear ribonucleoprotein C (C1/C2)	Nucleus	other
HNRNPF	3185	heterogeneous nuclear ribonucleoprotein F	Nucleus	other
HNRNPH3	3189	heterogeneous nuclear ribonucleoprotein H3 (2H9)	Nucleus	other
HOOK1	51361	hook microtubule-tethering protein 1	Cytoplasm	other
HSP90AA1	3320	heat shock protein 90kDa alpha (cytosolic), class A member 1	Cytoplasm	enzyme
HSP90AB1	3326	heat shock protein 90kDa alpha (cytosolic), class B member 1	Cytoplasm	enzyme
HSPA1L	3305	heat shock 70kDa protein 1-like	Cytoplasm	other
HSPA4	3308	heat shock 70kDa protein 4	Cytoplasm	other
HSPA5	3309	heat shock 70kDa protein 5 (glucose-regulated protein, 78kDa)	Cytoplasm	enzyme
IFNAR2	3455	interferon (alpha, beta and omega)	Plasma	transmembrane

		receptor 2	Membrane	receptor
ILF3	3609	interleukin enhancer binding factor 3, 90kDa	Nucleus	transcription regulator
ITGB3	3690	integrin, beta 3 (platelet glycoprotein IIIa, antigen CD61)	Plasma Membrane	transmembrane receptor
ITIH3	3699	inter-alpha-trypsin inhibitor heavy chain 3	Extracellular Space	other
JAG1	182	jagged 1	Extracellular Space	growth factor
KAT5	10524	K(lysine) acetyltransferase 5	Nucleus	transcription regulator
KIF1B	23095	kinesin family member 1B	Cytoplasm	transporter
KIF3B	9371	kinesin family member 3B	Cytoplasm	transporter
KNG1	3827	kininogen 1	Extracellular Space	other
KPNA1	3836	karyopherin alpha 1 (importin alpha 5)	Nucleus	transporter
KPNA3	3839	karyopherin alpha 3 (importin alpha 4)	Nucleus	transporter
KPNB1	3837	karyopherin (importin) beta 1	Nucleus	transporter
KRT19	3880	keratin 19, type I	Cytoplasm	other
KRT8	3856	keratin 8, type II	Cytoplasm	other
KTN1	3895	kinectin 1 (kinesin receptor)	Plasma Membrane	transmembrane receptor
LAMB1	3912	laminin, beta 1	Extracellular Space	other
LAMB2	3913	laminin, beta 2 (laminin S)	Extracellular Space	enzyme
LMNA	4000	lamin A/C	Nucleus	other
LRRC45	201255	leucine rich repeat containing 45	Cytoplasm	other
LTBP3	4054	latent transforming growth factor beta binding protein 3	Extracellular Space	other
LUC7L3	51747	LUC7-like 3 pre-mRNA splicing factor	Nucleus	other
MATR3	9782	matrin 3	Nucleus	other
MDFI	4188	MyoD family inhibitor	Cytoplasm	other
MDM2	4193	MDM2 proto-oncogene	Cytoplasm	other
MED4	29079	mediator complex subunit 4	Nucleus	transcription regulator
MISP	126353	mitotic spindle positioning	Plasma Membrane	other
MLPH	79083	melanophilin	Cytoplasm	other
MRPL20	55052	mitochondrial ribosomal protein L20	Cytoplasm	other
MYCBP2	23077	MYC binding protein 2, E3 ubiquitin protein ligase	Nucleus	enzyme
MYH14	79784	myosin, heavy chain 14, non-muscle	Extracellular Space	other
MYH9	4627	myosin, heavy chain 9, non-muscle	Cytoplasm	enzyme

NECAB2	54550	N-terminal EF-hand calcium binding protein 2	Cytoplasm	other
NEFH	4744	neurofilament, heavy polypeptide	Cytoplasm	other
NFKBIA	4792	nuclear factor of kappa light polypeptide gene enhancer in B-cells inhibitor, alpha	Cytoplasm	transcription regulator
NFKBIB	4793	nuclear factor of kappa light polypeptide gene enhancer in B-cells inhibitor, beta	Nucleus	transcription regulator
NID1	4811	nidogen 1	Extracellular Space	other
NIPSNAP1	8508	nipsnap homolog 1 (C. elegans)	Cytoplasm	enzyme
NKAPL	222698	NFKB activating protein-like	Other	other
NME3	4832	NME/NM23 nucleoside diphosphate kinase 3	Cytoplasm	kinase
NOC2L	26155	NOC2-like nucleolar associated transcriptional repressor	Nucleus	transcription regulator
NRBP1	29959	nuclear receptor binding protein 1	Nucleus	kinase
NUMA1	4926	nuclear mitotic apparatus protein 1	Nucleus	other
NUP50	10762	nucleoporin 50kDa	Nucleus	transporter
OPTN	10133	optineurin	Cytoplasm	other
PABPC1	26986	poly(A) binding protein, cytoplasmic 1	Cytoplasm	translation regulator
PAIP1	10605	poly(A) binding protein interacting protein 1	Cytoplasm	translation regulator
PCBP2	5094	poly(rC) binding protein 2	Nucleus	other
PCM1	5108	pericentriolar material 1	Cytoplasm	other
PCNT	5116	pericentrin	Cytoplasm	other
PDCD6IP	10015	programmed cell death 6 interacting protein	Cytoplasm	other
PDE4DIP	9659	phosphodiesterase 4D interacting protein	Cytoplasm	enzyme
PGK1	5230	phosphoglycerate kinase 1	Cytoplasm	kinase
PHAX	51808	phosphorylated adaptor for RNA export	Cytoplasm	other
PHC2	1912	polyhomeotic homolog 2 (Drosophila)	Nucleus	other
PHLDB3	653583	pleckstrin homology-like domain, family B, member 3	Other	other
PIAS1	8554	protein inhibitor of activated STAT, 1	Nucleus	transcription regulator
PIAS3	10401	protein inhibitor of activated STAT, 3	Nucleus	transcription regulator
PKM	5315	pyruvate kinase, muscle	Cytoplasm	kinase
PLS3	5358	plastin 3	Cytoplasm	other
PPIA	5478	peptidylprolyl isomerase A (cyclophilin A)	Cytoplasm	enzyme
PPP1R3E	90673	protein phosphatase 1, regulatory	Other	other

		subunit 3E		
PPRC1	23082	peroxisome proliferator-activated receptor gamma, coactivator-related 1	Nucleus	transcription regulator
PRDX6	9588	peroxiredoxin 6	Cytoplasm	enzyme
PRKRA	8575	protein kinase, interferon-inducible double stranded RNA dependent activator	Cytoplasm	other
PRMT5	10419	protein arginine methyltransferase 5	Cytoplasm	enzyme
PSMD1	5707	proteasome 26S subunit, non-ATPase 1	Cytoplasm	other
PSMD13	5719	proteasome 26S subunit, non-ATPase 13	Cytoplasm	peptidase
PTBP1	5725	polypyrimidine tract binding protein 1	Nucleus	enzyme
RAI14	26064	retinoic acid induced 14	Nucleus	transcription regulator
RASSF7	8045	Ras association (RalGDS/AF-6) domain family (N-terminal) member 7	Other	other
RFWD2	64326	ring finger and WD repeat domain 2, E3 ubiquitin protein ligase	Cytoplasm	enzyme
RILPL2	196383	Rab interacting lysosomal protein-like 2	Cytoplasm	other
RLIM	51132	ring finger protein, LIM domain interacting	Nucleus	enzyme
RNASET2	8635	ribonuclease T2	Cytoplasm	enzyme
RNF125	54941	ring finger protein 125, E3 ubiquitin protein ligase	Other	other
RPS20	6224	ribosomal protein S20	Cytoplasm	other
SCLT1	132320	sodium channel and clathrin linker 1	Plasma Membrane	transporter
SCMH1	22955	sex comb on midleg homolog 1 (Drosophila)	Nucleus	transcription regulator
SCRIB	23513	scribbled planar cell polarity protein	Cytoplasm	other
SDCCAG8	10806	serologically defined colon cancer antigen 8	Cytoplasm	other
SEC31A	22872	SEC31 homolog A, COPII coat complex component	Cytoplasm	other
SERPIND1	3053	serpin peptidase inhibitor, clade D (heparin cofactor), member 1	Extracellular Space	other
SET	6418	SET nuclear proto-oncogene	Nucleus	phosphatase
SIAH2	6478	siah E3 ubiquitin protein ligase 2	Nucleus	transcription regulator
SMARCB1	6598	SWI/SNF related, matrix associated, actin dependent regulator of chromatin, subfamily b, member 1	Nucleus	transcription regulator
SPDL1	54908	spindle apparatus coiled-coil protein 1	Nucleus	other
SPG7	6687	spastic paraplegia 7 (pure and	Cytoplasm	peptidase

		complicated autosomal recessive)		
SPTAN1	6709	spectrin, alpha, non-erythrocytic 1	Plasma Membrane	other
SPTBN2	6712	spectrin, beta, non-erythrocytic 2	Cytoplasm	other
SQSTM1	8878	sequestosome 1	Cytoplasm	transcription regulator
SRFBP1	153443	serum response factor binding protein 1	Nucleus	other
SRSF11	9295	serine/arginine-rich splicing factor 11	Nucleus	other
SSB	6741	Sjogren syndrome antigen B (autoantigen La)	Nucleus	enzyme
STAT2	6773	signal transducer and activator of transcription 2, 113kDa	Nucleus	transcription regulator
STAT3	6774	signal transducer and activator of transcription 3 (acute-phase response factor)	Nucleus	transcription regulator
SYBU	55638	syntabulin (syntaxin-interacting)	Other	other
SYNE1	23345	spectrin repeat containing, nuclear envelope 1	Nucleus	other
SYNE2	23224	spectrin repeat containing, nuclear envelope 2	Nucleus	other
TAB2	23118	TGF-beta activated kinase 1/MAP3K7 binding protein 2	Cytoplasm	other
TAF15	8148	TAF15 RNA polymerase II, TATA box binding protein (TBP)-associated factor, 68kDa	Nucleus	other
TAX1BP1	8887	Tax1 (human T-cell leukemia virus type I) binding protein 1	Cytoplasm	other
TCF7L2	6934	transcription factor 7-like 2 (T-cell specific, HMG-box)	Nucleus	transcription regulator
TMEFF2	23671	transmembrane protein with EGF-like and two follistatin-like domains 2	Cytoplasm	other
TMEM57	55219	transmembrane protein 57	Nucleus	other
TMEM74	157753	transmembrane protein 74	Other	other
TNFRSF10B	8795	tumor necrosis factor receptor superfamily, member 10b	Plasma Membrane	transmembrane receptor
TNIP1	10318	TNFAIP3 interacting protein 1	Nucleus	other
TOM1L1	10040	target of myb1 like 1 membrane trafficking protein	Cytoplasm	other
TOX4	9878	TOX high mobility group box family member 4	Nucleus	other
TP53BP2	7159	tumor protein p53 binding protein 2	Nucleus	other
TRAF4	9618	TNF receptor-associated factor 4	Cytoplasm	other
TRAFD1	10906	TRAF-type zinc finger domain containing 1	Other	other
TRIM21	6737	tripartite motif containing 21	Nucleus	enzyme
TRIM62	55223	tripartite motif containing 62	Cytoplasm	enzyme

TRIP11	9321	thyroid hormone receptor interactor 11	Cytoplasm	transcription regulator
TSG101	7251	tumor susceptibility 101	Cytoplasm	transcription regulator
TTC1	7265	tetratricopeptide repeat domain 1	Cytoplasm	other
TUBB	203068	tubulin, beta class I	Cytoplasm	other
TXNDC9	10190	thioredoxin domain containing 9	Cytoplasm	other
TYK2	7297	tyrosine kinase 2	Plasma Membrane	kinase
UACA	55075	uveal autoantigen with coiled-coil domains and ankyrin repeats	Cytoplasm	other
UBE2I	7329	ubiquitin-conjugating enzyme E2I	Nucleus	enzyme
UBXN2A	165324	UBX domain protein 2A	Other	other
VIM	7431	vimentin	Cytoplasm	other
VPS11	55823	vacuolar protein sorting 11 homolog (S. cerevisiae)	Cytoplasm	transporter
VPS37A	137492	vacuolar protein sorting 37 homolog A (S. cerevisiae)	Cytoplasm	other
WWC1	23286	WW and C2 domain containing 1	Cytoplasm	transcription regulator
XPO1	7514	exportin 1	Nucleus	transporter
ZBTB17	7709	zinc finger and BTB domain containing 17	Nucleus	transcription regulator
ZBTB8OS	339487	zinc finger and BTB domain containing 8 opposite strand	Cytoplasm	other
ZCCHC17	51538	zinc finger, CCHC domain containing 17	Nucleus	other
ZNF135	7694	zinc finger protein 135	Nucleus	transcription regulator
ZNF252P-AS1	286103	ZNF252P antisense RNA 1	Other	other
ZNF688	146542	zinc finger protein 688	Other	other
