# Supplementary material for: An Integrative Analysis Reveals a Central Role of P53 Activation via MDM2 in Zika Virus Infection Induced Cell Death
Source: Front Cell Infect Microbiol. 2017 Jul 20;7:327. doi: 10.3389/fcimb.2017.00327 (PMC5517408; doi:10.3389/fcimb.2017.00327)
Supplement: Table S2 — Microcephaly-associated proteins in the human genome. [file Table2.PDF]

Table S2 Microcephaly-associated proteins in the human genome

Human Symbol	Gene ID	Entrez Gene Name	Location	Type(s)
ABCD4	5826	ATP-binding cassette, sub-family D (ALD), member 4	Cytoplasm	transporter
ACVRL1	94	activin A receptor type II-like 1	Plasma Membrane	kinase
AKT3	10000	v-akt murine thymoma viral oncogene homolog 3	Cytoplasm	kinase
AP4E1	23431	adaptor-related protein complex 4, epsilon 1 subunit	Cytoplasm	other
APOE	348	apolipoprotein E	Extracellular Space	transporter
ARFGEF2	10564	ADP-ribosylation factor guanine nucleotide-exchange factor 2 (brefeldin A-inhibited)	Cytoplasm	other
ASPM	259266	abnormal spindle microtubule assembly	Nucleus	other
ATP1A3	478	ATPase, Na+/K+ transporting, alpha 3 polypeptide	Plasma Membrane	transporter
ATR	545	ATR serine/threonine kinase	Nucleus	kinase
BCOR	54880	BCL6 corepressor	Nucleus	transcription regulator
BNC2	54796	basonuclin 2	Nucleus	other
BRCA1	672	breast cancer 1, early onset	Nucleus	transcription regulator
BRWD3	254065	bromodomain and WD repeat domain containing 3	Other	other
CASC5	57082	cancer susceptibility candidate 5	Nucleus	other
CASK	8573	calcium/calmodulin-dependent serine protein kinase (MAGUK family)	Plasma Membrane	kinase
CDK5RAP2	55755	CDK5 regulatory subunit associated protein 2	Cytoplasm	other
CDK6	1021	cyclin-dependent kinase 6	Nucleus	kinase
CDKL5	6792	cyclin-dependent kinase-like 5	Nucleus	kinase
CENPE	1062	centromere protein E, 312kDa	Nucleus	other
CENPJ	55835	centromere protein J	Nucleus	transcription regulator
CEP135	9662	centrosomal protein 135kDa	Cytoplasm	other
CEP152	22995	centrosomal protein 152kDa	Cytoplasm	other
CHEK1	1111	checkpoint kinase 1	Nucleus	kinase
CHRD	8646	chordin	Extracellular Space	other
CKAP2L	150468	cytoskeleton associated protein 2-like	Cytoplasm	other
CLP1	10978	cleavage and polyadenylation factor I subunit 1	Nucleus	other
CLPB	81570	ClpB homolog, mitochondrial AAA ATPase chaperonin	Nucleus	transcription regulator

COL2A1	1280	collagen, type II, alpha 1	Extracellular Space	other
COX20	116228	COX20 cytochrome c oxidase assembly factor	Cytoplasm	other
COX7B	1349	cytochrome c oxidase subunit VIIb	Cytoplasm	enzyme
CPLX1	10815	complexin 1	Plasma Membrane	transporter
CRIPT	9419	cysteine-rich PDZ-binding protein	Cytoplasm	other
CTBP1	1487	C-terminal binding protein 1	Nucleus	enzyme
CTNNB1	1499	catenin (cadherin-associated protein), beta 1, 88kDa	Nucleus	transcription regulator
DCTN5	84516	dynactin 5 (p25)	Cytoplasm	other
DDB2	1643	damage-specific DNA binding protein 2, 48kDa	Nucleus	other
DEAF1	10522	DEAF1 transcription factor	Nucleus	transcription regulator
DHCR7	1717	7-dehydrocholesterol reductase	Cytoplasm	enzyme
DKK1	22943	dickkopf WNT signaling pathway inhibitor 1	Extracellular Space	growth factor
DYRK1A	1859	dual-specificity tyrosine-(Y)-phosphorylation regulated kinase 1A	Nucleus	kinase
EFTUD2	9343	elongation factor Tu GTP binding domain containing 2	Nucleus	enzyme
EIF2S3	1968	eukaryotic translation initiation factor 2, subunit 3 gamma, 52kDa	Cytoplasm	translation regulator
ENG	2022	endoglin	Plasma Membrane	transmembrane receptor
EOMES	8320	eomesodermin	Nucleus	transcription regulator
ERCC1	2067	excision repair cross-complementation group 1	Nucleus	enzyme
ERCC2	2068	excision repair cross-complementation group 2	Nucleus	enzyme
ERCC3	2071	excision repair cross-complementation group 3	Nucleus	enzyme
ERCC4	2072	excision repair cross-complementation group 4	Nucleus	enzyme
ERCC5	2073	excision repair cross-complementation group 5	Nucleus	enzyme
ESCO2	157570	establishment of sister chromatid cohesion N-acetyltransferase 2	Nucleus	enzyme
EVC2	132884	Ellis van Creveld syndrome 2	Extracellular Space	other
EYA1	2138	EYA transcriptional coactivator and phosphatase 1	Nucleus	phosphatase
FAR1	84188	fatty acyl CoA reductase 1	Cytoplasm	enzyme
FEZF2	55079	FEZ family zinc finger 2	Other	transcription regulator

FGFRL1	53834	fibroblast growth factor receptor-like 1	Plasma Membrane	transmembrane receptor
FKTN	2218	fukutin	Extracellular Space	other
FOXG1	2290	forkhead box G1	Nucleus	transcription regulator
FOXH1	8928	forkhead box H1	Nucleus	transcription regulator
FRS2	10818	fibroblast growth factor receptor substrate 2	Plasma Membrane	other
GJA1	2697	gap junction protein, alpha 1, 43kDa	Plasma Membrane	transporter
GLI2	2736	GLI family zinc finger 2	Nucleus	transcription regulator
HDAC8	55869	histone deacetylase 8	Nucleus	transcription regulator
HES1	3280	hes family bHLH transcription factor 1	Nucleus	transcription regulator
HESX1	8820	HESX homeobox 1	Nucleus	transcription regulator
HMGB3	3149	high mobility group box 3	Nucleus	other
HNRNPU	3192	heterogeneous nuclear ribonucleoprotein U (scaffold attachment factor A)	Nucleus	transporter
HSF4	3299	heat shock transcription factor 4	Nucleus	transcription regulator
MDM2	4193	MDM2 proto-oncogene	Cytoplasm	other
IER3IP1	51124	immediate early response 3 interacting protein 1	Cytoplasm	other
IGF1R	3480	insulin-like growth factor 1 receptor	Plasma Membrane	transmembrane receptor
IGFALS	3483	insulin-like growth factor binding protein, acid labile subunit	Extracellular Space	other
ITGAV	3685	integrin, alpha V	Plasma Membrane	ion channel
KAT6A	7994	K(lysine) acetyltransferase 6A	Nucleus	enzyme
KATNB1	10300	katanin p80 (WD repeat containing) subunit B 1	Cytoplasm	enzyme
KIF11	3832	kinesin family member 11	Nucleus	other
KIF14	9928	kinesin family member 14	Cytoplasm	enzyme
KIF20B	9585	kinesin family member 20B	Nucleus	enzyme
KIF21A	55605	kinesin family member 21A	Cytoplasm	other
KLF2	10365	Kruppel-like factor 2	Nucleus	transcription regulator
LETM1	3954	leucine zipper-EF-hand containing transmembrane protein 1	Cytoplasm	other
LIAS	11019	lipoic acid synthetase	Cytoplasm	enzyme
LIG3	3980	ligase III, DNA, ATP-dependent	Nucleus	enzyme

LIG4	3981	ligase IV, DNA, ATP-dependent	Nucleus	enzyme
LIM2	3982	lens intrinsic membrane protein 2, 19kDa	Plasma Membrane	other
LMBRD1	55788	LMBR1 domain containing 1	Cytoplasm	other
LMNB1	4001	lamin B1	Nucleus	other
MAP3K3	4215	mitogen-activated protein kinase kinase kinase 3	Cytoplasm	kinase
MBD5	55777	methyl-CpG binding domain protein 5	Nucleus	other
MCPH1	79648	microcephalin 1	Nucleus	other
MECP2	4204	methyl CpG binding protein 2	Nucleus	transcription regulator
MED1	5469	mediator complex subunit 1	Nucleus	transcription regulator
MED17	9440	mediator complex subunit 17	Nucleus	transcription regulator
MESP1	55897	mesoderm posterior bHLH transcription factor 1	Nucleus	transcription regulator
MFSD2A	84879	major facilitator superfamily domain containing 2A	Plasma Membrane	transporter
MFSD2A	407975	major facilitator superfamily domain containing 2A	Plasma Membrane	transporter
MIR17HG	57496	miR-17-92 cluster host gene	Other	other
MKL2	25974	MKL/myocardin-like 2	Nucleus	transcription regulator
MMACHC	4361	methylmalonic aciduria (cobalamin deficiency) cblC type, with homocystinuria	Cytoplasm	other
MRE11A	6307	MRE11 homolog A, double strand break repair nuclease	Nucleus	enzyme
MSMO1	25902	methylsterol monooxygenase 1	Cytoplasm	enzyme
MTHFD1L	4548	methylenetetrahydrofolate dehydrogenase (NADP+ dependent) 1-like	Cytoplasm	enzyme
MTR	4552	5-methyltetrahydrofolate-homocysteine methyltransferase	Cytoplasm	enzyme
MTRR	4613	5-methyltetrahydrofolate-homocysteine methyltransferase reductase	Cytoplasm	enzyme
MYCN	8260	v-myc avian myelocytomatosis viral oncogene neuroblastoma derived homolog	Nucleus	transcription regulator
NAA10	4683	N(alpha)-acetyltransferase 10, NatA catalytic subunit	Nucleus	enzyme
NBN	644861	nibrin	Nucleus	other
NBPF13P	284565	neuroblastoma breakpoint family, member 13, pseudogene	Other	other
NBPF15	401967	neuroblastoma breakpoint family, member 15	Other	other
NBPF17P	441908	neuroblastoma breakpoint family,	Other	other

		member 17, pseudogene		
NBPF18P	205655	neuroblastoma breakpoint family, member 18, pseudogene	Other	other
NBPF21P	285622	neuroblastoma breakpoint family, member 21, pseudogene	Other	other
NBPF22P	101929780	neuroblastoma breakpoint family, member 22, pseudogene	Other	other
NBPF25P	343381	neuroblastoma breakpoint family, member 25, pseudogene	Other	other
NBPF2P	84224	neuroblastoma breakpoint family, member 2, pseudogene	Other	other
NBPF3	148545	neuroblastoma breakpoint family, member 3	Other	other
NBPF4/NBPF6	653149	neuroblastoma breakpoint family, member 4	Other	other
NBPF7	343505	neuroblastoma breakpoint family, member 7	Other	other
NDE1	54820	nudE neurodevelopment protein 1	Nucleus	other
NHEJ1	79840	nonhomologous end-joining factor 1	Nucleus	other
NIPBL	25836	Nipped-B homolog (Drosophila)	Nucleus	transcription regulator
NLGN3	54413	neuroligin 3	Plasma Membrane	enzyme
NLGN4X	57502	neuroligin 4, X-linked	Plasma Membrane	enzyme
NRXN1	9378	neurexin 1	Plasma Membrane	transporter
NSDHL	50814	NAD(P) dependent steroid dehydrogenase-like	Cytoplasm	enzyme
OTULIN	90268	OTU deubiquitinase with linear linkage specificity	Cytoplasm	peptidase
OTX2	5015	orthodenticle homeobox 2	Nucleus	transcription regulator
PAH	5053	phenylalanine hydroxylase	Cytoplasm	enzyme
PAX6	5080	paired box 6	Nucleus	transcription regulator
PCLO	27445	piccolo presynaptic cytomatrix protein	Cytoplasm	transporter
PCNT	5116	pericentrin	Cytoplasm	other
PDCD10	11235	programmed cell death 10	Cytoplasm	other
PDS5B	23047	PDS5 cohesin associated factor B	Nucleus	other
PEX2	5828	peroxisomal biogenesis factor 2	Cytoplasm	other
PGAP1	80055	post-GPI attachment to proteins 1	Cytoplasm	enzyme
PHC1	1911	polyhomeotic homolog 1 (Drosophila)	Nucleus	transcription regulator
PHGDH	26227	phosphoglycerate dehydrogenase	Cytoplasm	enzyme
PHOX2A	401	paired-like homeobox 2a	Nucleus	transcription regulator

PITX3	5309	paired-like homeodomain 3	Nucleus	transcription regulator
PLK4	10733	polo-like kinase 4	Cytoplasm	kinase
PNKP	11284	polynucleotide kinase 3'-phosphatase	Nucleus	kinase
POLH	5429	polymerase (DNA directed), eta	Nucleus	enzyme
POMT1	10585	protein-O-mannosyltransferase 1	Cytoplasm	enzyme
POMT2	29954	protein-O-mannosyltransferase 2	Cytoplasm	enzyme
PPP1R15B	84919	protein phosphatase 1, regulatory subunit 15B	Cytoplasm	phosphatase
PQBP1	10084	polyglutamine binding protein 1	Nucleus	transcription regulator
PRICKLE1	144165	prickle homolog 1	Nucleus	other
PRRX1	5396	paired related homeobox 1	Nucleus	transcription regulator
PTCHD1	139411	patched domain containing 1	Plasma Membrane	other
PTPN11	5781	protein tyrosine phosphatase, non-receptor type 11	Cytoplasm	phosphatase
PTPRJ	5795	protein tyrosine phosphatase, receptor type, J	Plasma Membrane	phosphatase
PTRH2	51651	peptidyl-tRNA hydrolase 2	Cytoplasm	enzyme
QARS	5859	glutaminyl-tRNA synthetase	Cytoplasm	enzyme
RAD21	5885	RAD21 cohesin complex component	Nucleus	transcription regulator
RAD50	10111	RAD50 homolog, double strand break repair protein	Nucleus	enzyme
RBBP8	5932	retinoblastoma binding protein 8	Nucleus	enzyme
RNASEH2A	10535	ribonuclease H2, subunit A	Nucleus	enzyme
RPL10	6134	ribosomal protein L10	Cytoplasm	other
RPS6KA3	6197	ribosomal protein S6 kinase, 90kDa, polypeptide 3	Cytoplasm	kinase
RPS6KA6	27330	ribosomal protein S6 kinase, 90kDa, polypeptide 6	Cytoplasm	kinase
RYK	6259	receptor-like tyrosine kinase	Plasma Membrane	kinase
SASS6	163786	SAS-6 centriolar assembly protein	Cytoplasm	other
SATB2	23314	SATB homeobox 2	Nucleus	transcription regulator
SET	6418	SET nuclear proto-oncogene	Nucleus	phosphatase
SFN	2810	stratifin	Cytoplasm	other
SHANK2	22941	SH3 and multiple ankyrin repeat domains 2	Plasma Membrane	other
SHH	6469	sonic hedgehog	Extracellular Space	peptidase
SLC25A19	60386	solute carrier family 25 (mitochondrial thiamine pyrophosphate carrier), member 19	Cytoplasm	transporter

SLC2A1	6513	solute carrier family 2 (facilitated glucose transporter), member 1	Plasma Membrane	transporter
SLC9A6	10479	solute carrier family 9, subfamily A (NHE6, cation proton antiporter 6), member 6	Plasma Membrane	transporter
SMAD2	4087	SMAD family member 2	Nucleus	transcription regulator
SMARCA4	6597	SWI/SNF related, matrix associated, actin dependent regulator of chromatin, subfamily a, member 4	Nucleus	transcription regulator
SMC1A	8243	structural maintenance of chromosomes 1A	Nucleus	transporter
SMC3	9126	structural maintenance of chromosomes 3	Nucleus	other
SNRPN	6638	small nuclear ribonucleoprotein polypeptide N	Nucleus	other
SNX3	8724	sorting nexin 3	Cytoplasm	transporter
SPATA5	166378	spermatogenesis associated 5	Cytoplasm	other
SSBP3	23648	single stranded DNA binding protein 3	Nucleus	transcription regulator
STAMBP	10617	STAM binding protein	Nucleus	enzyme
STIL	6491	SCL/TAL1 interrupting locus	Nucleus	other
SUFU	51684	suppressor of fused homolog (Drosophila)	Nucleus	transcription regulator
TAF2	6873	TAF2 RNA polymerase II, TATA box binding protein (TBP)-associated factor, 150kDa	Nucleus	transcription regulator
TBX2	6909	T-box 2	Nucleus	transcription regulator
TBX4	9496	T-box 4	Nucleus	transcription regulator
TCOF1	6949	Treacher Collins-Franceschetti syndrome 1	Nucleus	transporter
TGIF1	7050	TGFB-induced factor homeobox 1	Nucleus	transcription regulator
THOC6	79228	THO complex 6	Nucleus	other
TMEM67	91147	transmembrane protein 67	Plasma Membrane	other
TRAPPC9	83696	trafficking protein particle complex 9	Plasma Membrane	other
TRMT10A	93587	tRNA methyltransferase 10A	Cytoplasm	other
TTN	7273	titin	Cytoplasm	kinase
TUBA1A	7846	tubulin, alpha 1a	Cytoplasm	other
TUBB	203068	tubulin, beta class I	Cytoplasm	other
TUBB3	10381	tubulin, beta 3 class III	Cytoplasm	other
TUBGCP4	27229	tubulin, gamma complex associated protein 4	Cytoplasm	other
TUBGCP6	85378	tubulin, gamma complex associated	Cytoplasm	other

		protein 6		
TWIST1	7291	twist family bHLH transcription factor 1	Nucleus	transcription regulator
UBE3A	7337	ubiquitin protein ligase E3A	Nucleus	enzyme
VPS13B	157680	vacuolar protein sorting 13 homolog B (yeast)	Nucleus	transporter
VRK1	7443	vaccinia related kinase 1	Nucleus	kinase
WDR62	284403	WD repeat domain 62	Nucleus	other
WDR73	84942	WD repeat domain 73	Extracellular Space	other
WHSC1	7468	Wolf-Hirschhorn syndrome candidate 1	Nucleus	enzyme
WIPF3	644150	WAS/WASL interacting protein family, member 3	Plasma Membrane	other
XPA	7507	xeroderma pigmentosum, complementation group A	Nucleus	other
XPC	7508	xeroderma pigmentosum, complementation group C	Nucleus	other
XPNPEP1	7511	X-prolyl aminopeptidase (aminopeptidase P) 1, soluble	Cytoplasm	peptidase
XRCC4	7518	X-ray repair complementing defective repair in Chinese hamster cells 4	Nucleus	other
ZEB2	9839	zinc finger E-box binding homeobox 2	Nucleus	transcription regulator
ZIC2	7546	Zic family member 2	Nucleus	transcription regulator
ZIC5	85416	Zic family member 5	Nucleus	transcription regulator
ZNF335	63925	zinc finger protein 335	Nucleus	other
ZNF592	9640	zinc finger protein 592	Nucleus	other
