# Supplementary material for: An Integrative Analysis Reveals a Central Role of P53 Activation via MDM2 in Zika Virus Infection Induced Cell Death
Source: Front Cell Infect Microbiol. 2017 Jul 20;7:327. doi: 10.3389/fcimb.2017.00327 (PMC5517408; doi:10.3389/fcimb.2017.00327)
Supplement: Supplementary file 11 [file DataSheet1.DOCX]

**Supplemental Information**

**Title: An integrative analysis reveals a central role of P53 activation via MDM2 in Zika virus infection induced cell death**

**Running title: ZIKV Capsid protein involves in MDM2-mediated P53 cell death pathway**

Yue Teng^1, 2, *, †^, Shufeng Liu^3, *^, Xiaocan Guo^4, *^, Shuxia Liu^5^, Yuan Jin ^6^, Tongtong He^1^, Dehua Bi^1^, Pei Zhang^7^, Baihan Lin^8^, Xiaoping An^1^, Dan Feng^9^, Zhiqiang Mi^1^, Yigang Tong^1, †^

*These authors contributed equally to this work.

1 State Key Laboratory of Pathogen and Biosecurity;

2 Beijing Institute of Microbiology and Epidemiology, Beijing 100071, China;

3 Center for Infectious Diseases, SRI International, Harrisonburg, VA 22802, USA;

4 Massachusetts Institute of Technology, 77 Massachusetts Avenue, Cambridge, MA 02139, USA;

5 College of Nuclear Science and Technology, Beijing Normal University, Beijing 100875, China;

6 Beijing Institute of Biotechnology, Beijing 100071, China;

7 Department of Neurobiology, Tongji Medical School, Huazhong University of Science and Technology, Wuhan 430030, China;

8 Computational Neuroscience Program, Department of Psychology, Physics, and Computer Science and Engineering; Institute for Protein Design, University of Washington, Seattle, WA 98195, USA;

9 Division of Standard Operational Management, Institute of Hospital Management, Chinese PLA General Hospital, Beijing 100853, China.

† Correspondence should be addressed to:

Yue Teng Ph.D., M.D., State Key Laboratory of Pathogen and Biosecurity, Beijing Institute of Microbiology and Epidemiology, 20 Dong-Da Street, Fengtai District, Beijing 100071, China. Telephone: +86-10-68164807; Fax: +86-10-68167357; E-mail: [yueteng@sklpb.org](mailto:yueteng@sklpb.org)

Yigang Tong Ph.D., State Key Laboratory of Pathogen and Biosecurity, Beijing Institute of Microbiology and Epidemiology, 20 Dong-Da Street, Fengtai District, Beijing 100071, China. Phone: 8610-68164807; Fax: 8610-68167357; E-mail: [tong.yigang@gmail.com](mailto:tong.yigang@gmail.com)

**Landscape effects in the function of ZIKV-related and microcephaly-associated molecular factors**

We aimed to predict the ZIKV-related human protein interaction network (see Methods for further details). Therefore, we investigated 248 human proteins that might interact with ZIKV proteins (Table S1) and subsequently explored their function and disease annotations in the ZIKV–human interactions protein network (Figure S1). The proteins known to be associated with *Flaviviridae* infection (CD209, DNM2, ERC1, IFNAR2, MDM2 and TUBB) were included within this list of the predicted proteins (Figure S3A). And similar to the multiple *Flaviviruses* in literature-curated interactions, the Capsid protein of ZIKV might interact with human MDM2 protein in datamining of the ZIKV-associated proteins. Importantly, associations between cell death (apoptosis, necrosis, and autophagy) and neurological disease were also observed, such as CASP8 (Caspase 8, apoptosis-related cysteine peptidase) and NFKBIA/B (nuclear factor of kappa light polypeptide gene enhancer in B-cells inhibitor alpha/beta), as shown in Figure S3A.

Reasoning that microcephaly-associated proteins in the human genome might provide insight into the mechanism responsible for ZVDs, we then performed a datamining analysis to identify the possible 212 proteins believed to encompass microcephaly (Table S2) and mapped their interaction network (Figure S2). The result of function mapping demonstrated that these proteins, which include HDAC8, HNRNPU, KLF2, MED17, SLC2A1, SLC9A6, SMAD2, and TUBB, were associated with viral infection and infection by RNA viruses, in addition to their relationship with brain disorders and cell death (Figure S3B). These results reveal that ZIKV infection can induce cell death in ZVD, and suggest that the viral infection-derived cell death in brain development or neuron differentiation could cause microcephaly.

**Gene ontology involved with ZIKV–human interaction proteins and microcephaly-associated human proteins**

Using GO and DAVID databases, the identified ZIKV–human interaction proteins and microcephaly-associated proteins were clustered into groups based on their molecular functions (Figure S4A and S4D), biological processes (Figure S4B and S4E), and cellular compartments (Figure S4C and S4F). When the ZIKV–human interaction proteins were sorted based on molecular function, they clustered into groups involved in enzyme binding (GO:0019899), RNA binding (GO:0003723), receptor binding (GO:0005102), and transcription factor binding (GO:0008134), all of which are associated with RNA virus regulation (Figure S4A). This analysis also grouped the proteins into four cell death-associated biological process classes (Figure S4B): programmed cell death (GO:0012501), apoptotic process (GO:0006915), regulation of cell death (GO:0010941), and regulation of programmed cell death (GO:0043067). Interestingly, three protein clusters, neuron part (GO:0097458), neuron projection (GO:0043005), and centrosome (GO:0005813) were further categorized into subcellular distributions according to cellular compartment and were associated with brain/head development (Figure S4C). This is consistent with previous findings in functional networks and implies that ZIKV infection could lead to cell death in neuron cells (Figure S3A).

The microcephaly-associated proteins encompassed extracellular, membrane, mitochondrial, and nuclear proteins. The functions of these molecules include ribonucleotide binding (GO:0032553, GO:0032549), purine ribonucleotide triphosphate binding (GO:0035639), and purine ribonucleotide binding (GO:0032550 and GO:0032555) (Figure S4D). The clusters within the biological processes included proteins that are involved in embryo development (GO:0009790), neurogenesis (GO:0022008), central nervous system development (GO:0007417), and the generation of neurons (GO:0048699), which are all related to brain development (Figure S4E). In addition, the cellular component classes of the microcephaly-associated proteins were mostly associated with chromatin, such as chromosome (GO:0005694), nuclear chromosome (GO:0000228), and chromatin (GO:0000785) (Figure S4F).

**Homology modeling and molecular docking**

We supplemented the amino acids (residues 98-104) of the structure for ZIKV-Capsid protein by homology modeling and we obtained the stable structure of the ZIKV Capsid protein (residues 25-104) in Figure S7A and S7B. Briefly, the updated ZIKV Capsid protein (residues 25-104) is buried by explicit water molecules and ions, respectively. The ionic concentration is 150mM. TIP3P is used to model water molecules. The protein-water complexes are performed 30000 steps energy minimization and then performed 80 nanoseconds (ns) MD simulation. The model is made by VMD (version 1.9) and MD simulation is performed by using NAMD (version 2.9) with the force file CHARMM at constant temperature of 310 K. The non-bonded Coulomb and van der Waals interactions are calculated with a cutoff using a switching function starting at 13 Å and reaching zero at 15 Å. The integration time step is 2 fs. Then, we focused on the amino acids residues 30-53, 74-97 and 81-104 in ZIKV-Capsid protein and docked with the MDM2, respectively. Then, the docking results showed that the MDM2 protein have strong interaction with the residues 74-97 of ZIKV-Capsid protein in Figure 3D and 3E, but does no interact with the residues 81-104 (Figure S7C) and 30-53 (Figure S7D), respectively. And we selected the peptide of ZIKV-Capsid (residues 30-53) as non-specific peptide for subsequent experiments. All MD simulation results were used to calculate the binding free energies, which were used to gain further insight into the binding interactions between the peptides and MDM2 protein. And the binding free energies are -242 (Residues: 74-97), -78.69 (Residues: 30-53) and -56.89 (Residues: 81-104) kcal/mol, respectively.

**Video**

The BALB/C mice were randomly divided into three experimental groups. Group A mice were un-injected as a blank control. In group B, the normal mice with tails marked black were injected non-specific peptide as the control group. In group C, the mice with tails marked red were injected with mimic peptide of ZIKV capsid protein as the treatment group. As shown in supplemental video 1, the mice in the group A blank control group demonstrated normal behavior of mice. These mice explored around in smooth and consecutive movements. The behavior of mice in the group B control group are very similar to those of the group A blank control group. Comparing to these two control groups, the treatment group injected with synthetic peptide of ZIKV capsid protein showed a significant impairment in behavioral capacities in supplemental video 2. These mice demonstrated jagged and discontinuous movements with limited exploration activities. These symptoms of inactive movement implied those of brain injury.

**Materials and Methods**

***Multiple sequence alignments***

To assess the genome-wide nucleotide and amino acid signatures of ZIKV, we retrieved full genome sequences (as of Feb 01, 2016) for it from the NCBI database genome browser. Fifteen human ZIKV genomes from the 2015 outbreak were aligned to obtain the consensus sequence for ZIKV proteins. The multiple sequence alignment software MAFFT was used to align the ZIKV genomes([Katoh and Standley, 2013](#_ENREF_1)).

***Widespread identification of ZIKV-associated proteins in the human genome***

In this study, we investigated protein mimicry using structural similarities from the Protein Data Bank (PDB, <http://www.rcsb.org>). We also performed a Basic Local Alignment Search Tool (BLAST; https://blast.ncbi.nlm.nih.gov/Blast.cgi) analysis of this against the PDB sequence entries. Additionally, protein BLAST analysis was performed for each consensus protein sequence in the ZIKV genomes from the 2015 outbreak to identify the closest proteins, which were then used to search the entire PDB for structurally similar proteins (defined as proteins with z-scores > 2.0). Default settings with a score cutoff of 40 bits and a sequence overlap cutoff of 50% were used. We then extracted only those structures that were from the genus *Flavivirus* in the family *Flaviviridae* from the above results. In this manuscript, we refer to these structurally similar proteins as ZIKV-related proteins. ZIKV protein sequences were mapped on protein sequences by using BLAST Alignment software to make homology search against a database containing all the sequences of the protein partners stored in UNIPROT (http://www.uniprot.org/), and then the results give matched pathogen proteins (E-score < 0.05) with links to UNIPROT IDs, which are the ZIKV-related proteins. In the further, we used the ZIKV-related proteins to catch and infer the viral-human protein by protein-protein interaction from viral protein sequence in different databases, publications or supported by distinct experimental procedures.

***Generation of a ZIKV-related human molecular interaction network***

To predict which human proteins may interact with ZIKV proteins, we sought to identify the human proteins that interact with ZIKV-related proteins during cellular processes. We examined known interactions between ZIKV-related proteins and human proteins, using data from UNIPROT, which contains literature-curated interactions between pairs of human proteins, to obtain a global host response during ZIKV infection. Proteins identified by recent studies as interacting with ZIKV proteins were also included in our network. The public human protein–protein interaction database HIPPIE (Human Integrated Protein-Protein Interaction reference)([Schaefer et al., 2012](#_ENREF_2)), which includes BioGrid, DIP, HPRD, IntAct, MINT, and BIND databases, was selected as the data source to identify the high-confidence interactions based on the UniProt database.

***Datamining of the microcephaly-associated proteins***

Datamining analysis of the microcephaly-associated proteins was performed using genes from the NCBI database (<http://www.ncbi.nlm.nih.gov/gene/?term=Microcephaly>) and DisGeNet (<http://www.disgenet.org/web/DisGeNET/menu/dbinfo>). The entire databases were downloaded and filtered to scan for microcephaly-associated genes. A protein interaction network was constructed by querying the human protein–protein interaction database STRING Version 10.0 ([http://string-db.org/](http://cbdm.mdc-berlin.de/tools/hippie/index.php)) with the list of microcephaly-associated proteins that were common to two or more studies([Szklarczyk et al., 2015](#_ENREF_3)).

**References**

Katoh, K., and Standley, D.M. (2013). MAFFT multiple sequence alignment software version 7: improvements in performance and usability. *Mol Biol Evol* 30**,** 772-780. doi:10.1093/molbev/mst010.

Schaefer, M.H., Fontaine, J.F., Vinayagam, A., Porras, P., Wanker, E.E., and Andrade-Navarro, M.A. (2012). HIPPIE: Integrating protein interaction networks with experiment based quality scores. *PLoS One* 7**,** e31826. doi:10.1371/journal.pone.0031826.

Szklarczyk, D., Franceschini, A., Wyder, S., Forslund, K., Heller, D., Huerta-Cepas, J., et al. (2015). STRING v10: protein-protein interaction networks, integrated over the tree of life. *Nucleic Acids Res* 43**,** D447-452. doi:10.1093/nar/gku1003.
